# Imaging-based method to quantify left ventricular diastolic pressures

**DOI:** 10.1093/ehjci/jeaf017

**Published:** 2025-01-16

**Authors:** Otto A Smiseth, Joao F Fernandes, Nobuyuki Ohte, Kazuaki Wakami, Erwan Donal, Espen W Remme, Pablo Lamata

**Affiliations:** Institute for Surgical Research, Division of Cardiovascular and Pulmonary Diseases, Oslo University Hospital, Rikshospitalet and University of Oslo, Sognsvannsveien 20, NO-0372 Oslo, Norway; School of Biomedical Engineering and Imaging Sciences, King’s College London, London, UK; Department of Cardiology, Nagoya City University Graduate School of Medical Sciences, Nagoya, Japan; Department of Cardiology, Nagoya City University Graduate School of Medical Sciences, Nagoya, Japan; Department of Cardiology, CHU Rennes and Inserm, LTSI, University of Rennes, Rennes, France; Institute for Surgical Research, Oslo University Hospital, Rikshospitalet, Oslo, Norway; The Intervention Centre, Oslo University Hospital, Rikshospitalet, Oslo, Norway; School of Biomedical Engineering and Imaging Sciences, King’s College London, London, UK

**Keywords:** echocardiography, diastole, diastolic pressure, filling pressure, heart failure, left ventricle, isovolumic relaxation time

## Abstract

**Aims:**

To establish an imaging-based method to quantify left ventricular (LV) diastolic pressures.

**Methods and results:**

In 115 patients suspected of coronary artery disease, LV pressure was measured by micromanometers and images by echocardiography. LV filling pressure was measured as LV pre-atrial contraction pressure (pre-A P_LV_). Based on previous observations, we hypothesized that pre-A P_LV_ approximates the sum of minimum P_LV_ and maximum transmitral pressure difference. Parameters used for pressure estimates included LV volumes and strain, left atrial strain, mitral flow velocities, systolic arterial cuff pressure, and body mass index. Minimum P_LV_ was estimated by predictors identified in a derivative cohort (*n* = 81). Mitral pressure difference was calculated by a simplified Navier–Stokes equation. Accuracy of estimates of minimum P_LV_, pre-A P_LV_, and end-diastolic P_LV_ was investigated in a testing cohort (*n* = 19). Patient-specific LV diastolic pressure curves were constructed by adjusting a reference curve according to pressure estimates at key diastolic events. There was good agreement between estimated and measured pre-A P_LV_: bias 0.0, limits of agreement < 3.1 mmHg (±1.96 SD). Estimated minimum P_LV_ and end-diastolic P_LV_ also showed good agreement with measured pressures. Furthermore, there was good agreement between measured and estimated LV diastolic pressure curves, quantified as mean LV diastolic pressure: bias 0.2, limits of agreement < 3.2 mmHg.

**Conclusion:**

The proposed non-invasive method provided estimates of minimum P_LV_, pre-A P_LV_, and end-diastolic P_LV_, each reflecting different features of diastolic function. Additionally, it provided an estimate of the LV diastolic pressure curve. Validation in larger populations with different phenotypes is necessary to determine the validity of the method in clinical practice.

## Graphical Abstract

**Graphical Abstract:**
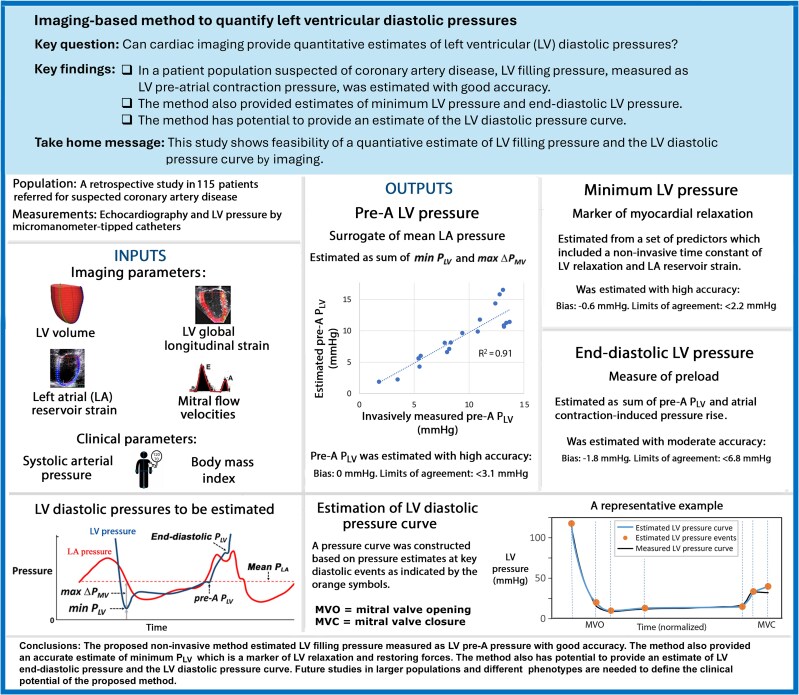

**See the editorial comment for this article ‘Predicting the diastolic left ventricular pressure curve non-invasively: how far can echo go?’, by F.A. Flachskampf and J.D. Thomas, https://doi.org/10.1093/ehjci/jeaf088.**

## Introduction

Left ventricular (LV) filling pressure is important as a diagnostic marker when evaluating patients for suspected heart failure and as a therapeutic target in patients with established heart failure and pulmonary vascular congestion. In clinical routine, echocardiography is the most widely used method for evaluating LV filling pressure, and a combination of several parameters can differentiate between normal and elevated LV filling pressure with good accuracy.^1–3^ Echocardiography, however, does not provide a quantitative estimate of LV filling pressure and does not differentiate between mean left atrial pressure (P_LA_) and end-diastolic LV pressure (P_LV_), which are used clinically as alternative parameters of filling pressure. This differentiation can be important in certain patients, as mean P_LA_ is a key factor in pulmonary vascular congestion, while LV end-diastolic pressure indicates preload and influences stroke volume. Furthermore, echocardiography does not provide an estimate of minimum P_LV_ that is an interesting parameter as it is related to LV relaxation and restoring forces.^4^ In the present study, we propose a method with potential to estimate the different components of LV diastolic pressure.

In 2012, we introduced a method to construct the LV systolic pressure curve and estimate myocardial work based on echocardiography.^5^ That method, however, does not provide an estimate of LV diastolic pressures, which is the focus of the present study. The main objective of the study was to develop a non-invasive method to provide quantitative estimates of LV diastolic pressures, and as a secondary objective, to investigate feasibility of constructing the LV diastolic pressure curve by an imaging approach. To ensure high accuracy in the pressure measurements, the study was conducted in patients investigated with high-fidelity LV pressure catheters.

## Methods

This study is divided into two parts: (i) estimation of LV filling pressure, and (ii) estimation of the LV diastolic pressure curve.

Clinical data originated from two studies previously published by the authors of this article (O.A.S., N.O., and K.W.).^6,7^ Both studies had approval from ethics committees and patient informed consent. The study included a total of 115 patients: 100 patients were used in the development of method to estimate LV diastolic pressure and 15 patients for development of method to construct a LV pressure curve (eight for defining a reference curve and seven for testing accuracy). Patients with acute coronary syndrome, primary valvular heart disease, atrial fibrillation, or intraventricular conduction disturbance were excluded.

### Part 1: non-invasive LV diastolic pressure

Part 1 of the study considered 100 patients who underwent cardiac catheterization due to suspected coronary artery disease (CAD).^6^ In all patients, LV pressures were measured by high-fidelity pressure sensors using micromanometer-tipped catheters. *Table 1* shows the characteristics of the patients. We used 81 patients as a derivative cohort and 19 patients as a testing cohort. As explained in more detail in Supplementary data online, *Data B*, subjects were assigned to each group sorted in sequential order of data acquisition, with the first 81 allocated to the derivative cohort and the last 19 to the testing cohort.

**Table 1:** Clinical characteristics (*n* = 100)

Male/female	80/20
Age (years)	66 ± 9
HR (beats/min)	65 ± 12
MAP (mmHg)	89 ± 10

	Preserved systolic function (EF ≥ 50%)	Systolic dysfunction (EF < 50%)
Number of patients	83	17
Atypical chest pain	10	0
CAD without MI	36	0
CAD with MI		
Anterior	28	8
Inferior	8	3
Combined	1	6

CAD, coronary artery disease; HR, heart rate; MAP, mean arterial pressure; MI, myocardial infarction.

Input data used for estimating LV diastolic pressures were echocardiographic measurements of LV end-diastolic and end-systolic volumes, LV and LA strain traces, mitral flow velocities, systolic brachial artery pressure by the cuff method, and body mass index (BMI).

#### Echocardiography

Echocardiography was performed within 2 h before catheterization using Aplio-80 or Aplio Artida SSH-880A (Toshiba Medical Co., Tokyo, Japan), with patients in left decubitus position.

Two-dimensional echocardiography was performed in the three apical views. Measurements of LV and LA dimensions were performed according to existing guidelines. LV global longitudinal strain (GLS) and LA reservoir and pump strains were measured by speckle tracking echocardiography using frame rates from 40 to 80 Hz. A maximum of two segments could be rejected due to suboptimal tracking when calculating GLS. LA reservoir and pump strains (LARS and LAPS, respectively) were calculated from recordings in apical four-chamber view. The measurements also included mitral early-diastolic (E) and atrial contraction-induced (A) mitral flow velocities, and early-diastolic mitral annular velocity (e′) as the average of velocities at the septal and lateral corners of the mitral annulus by pulsed Doppler echocardiography.

#### Measurement of LV pressure

LV pressure was measured with micromanometer-tipped catheters with a lumen for contrast injection (SPC-454D, Millar Instrument Co., Houston, TX, USA) and via the same lumen absolute LV pressure was measured by an external conventional manometer. All pressure transducers were calibrated with a mercury manometer. Pressure-zero was set at mid-chest level. To adjust for electronic drifting and hydrostatic effects on pressure as result of changes in catheter position, the micromanometer was zero-referenced at the end of diastasis against pressure recorded via the fluid-lumen inside the pressure catheter. Measurements were done before injection of contrast material.

**Figure 1:**
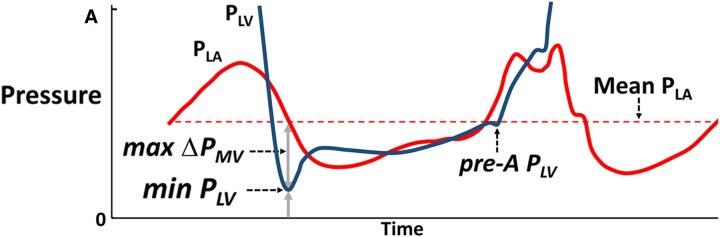
Schematic illustration of the principle that LV pre-atrial contraction pressure (pre-A P_LV_) was estimated as the sum of minimum LV pressure (min P_LV_) and the maximum early-diastolic atrio-ventricular pressure difference (max ΔP_MV_). The horizontal, dashed line represents mean left atrial pressure (mean P_LA_).

LV pressure (P_LV_) measurements included minimum P_LV_, pre-A P_LV_, and end-diastolic P_LV_ (*Figure 1*). As parameter of LV relaxation, we calculated the time constant of LV isovolumic pressure decay (τ) according to Weiss *et al*.^8^ The values of minimum P_LV_, pre-A P_LV_, and end-diastolic P_LV_ were calculated as an average from 10 consecutive heart cycles in each patient, whereas all the remaining pressure and echocardiography metrics were obtained as the median of 3 consecutive beats, recorded during breath holding at end-expiration. In the derivative cohort, systolic pressure was measured as peak LV pressure and in the testing cohort as systolic brachial artery pressure using the cuff method. Supplementary data online, *Data F* shows how errors in estimates of LV systolic pressure influence our diastolic pressure estimates. We found that the estimates were reasonably robust to 20% error in measurements in systolic pressure.

The analysis of echocardiographic data was performed blindly to the measured pressures.

##### Definition of LV filling pressure

In the present study, the term LV filling pressure refers to mean P_LA_ that was approximated as pre-A P_LV_.^1^ The validity of this approximation was confirmed in previous studies.^9,10^  *Figure 1* illustrates the different LV diastolic pressure components.

##### Method for estimating LV filling pressure

The method is built on three principles, as explained next.

The first principle is that mean pressure in the left atrium (P_LA_) can be approximated as the sum of minimum P_LV_ and maximum early-diastolic transmitral pressure difference (max ΔP_MV_), as illustrated in *Figure 1*. This principle is supported by several studies in conscious and anaesthetized dogs under a wide range of haemodynamic conditions, including congestive heart failure (HF), as summarized in Supplementary data online, *Data A*, which also explains the physical rationale for the principle. We used LV pre-A pressure as a substitute for mean LA pressure as described in Eq. (1).


(1) $$\begin{array}{c} \mathrm{LV}\;\mathrm{filling}\;\mathrm{pressure}=\mathrm{pre}\text{-}A\;P_{\mathrm{LV}}\approx \min\;P_{\mathrm{LV}}+\max\;P_{\mathrm{MV}} \end{array}\;$$

The second principle used in the estimation of LV filling pressure is that minimum P_LV_ can be estimated by a linear model from a set of biomarkers learned from analysis of the derivative cohort. A total of 10 candidate variables were explored (presented in Supplementary data online, *Data B*), and the final optimized model consisted of four independent predictors of minimum P_LV_ (see *Table 2*). These four predictors of minimum P_LV_ were incorporated in a multivariable linear regression model [Eq. (2)]:


(2) $$\begin{array}{c} \min\;P_{\mathrm{LV}}\approx -6.885+0.025\cdot P_{\mathrm{sys}}+0.243\cdot \mathrm{BMI}-0.097\cdot \mathrm{LARS}+0.158\cdot \tau \end{array}$$

**Table 2:** Independent predictors of minimum P_LV_

Independent predictors of minimum P_LV_	
Time constant τ of LV relaxation: *r* = 0.71	Left atrial reservoir strain: *r* = 0.65
Body mass index: *r* = 0.37	Systolic pressure: *r* = 0.23

**Figure 2:**
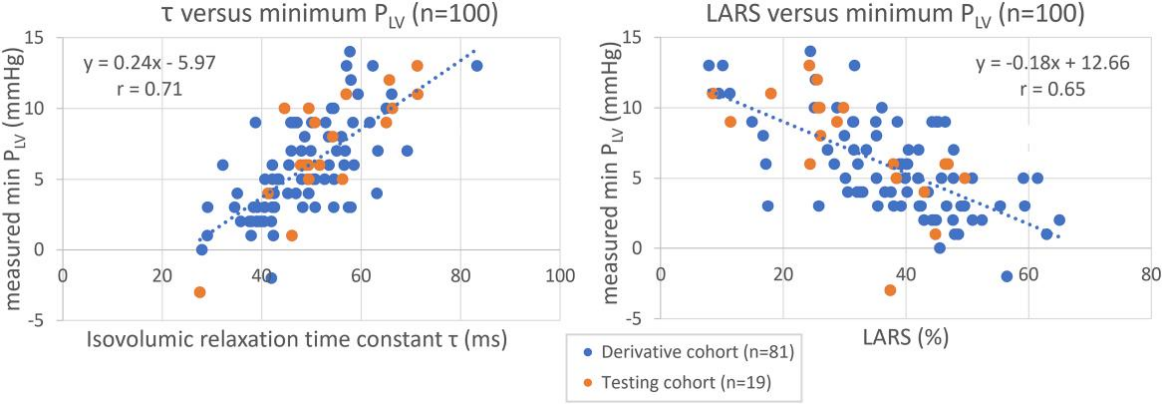
Association between measured minimum P_LV_ (min P_LV_) and LV isovolumic relaxation time constant (τ; left) and LA reservoir strain (LARS; right). Data from the full cohort of 100 patients are shown (derivative cohort, *n* = 81, and testing cohort, *n* = 19). Regression data for the derivative cohort are indicated.

The strongest predictors of minimum P_LV_ were the time constant of LV isovolumic relaxation (τ) and LA reservoir strain (LARS) (*Figure 2* and *Table 2*). Systolic pressure (P_sys_) and BMI were weaker, but statistically significant predictors (see Supplementary data online, *Data B*). A sensitivity analysis on errors of inputs for estimation of minimum P_LV_ is presented in Supplementary data online, *Data F*.

The third and last principle is that maximum ΔP_MV_ can be computed by a simplification of the Navier–Stokes momentum conservation equation. As such, the peak early-diastolic transmitral pressure difference is computed as:


(3) $$\begin{array}{c} P_{\mathrm{MV}}\approx \frac{\rho \;}{2}\left(v_{\mathrm{MV}}^{2}+\frac{\;Q\;v_{\mathrm{MV}}^{2}}{\Delta Q}\right)\; \end{array}$$

using the transmitral flow velocity (*v*_MV_), the flow rate (*Q*), and its increment in time (Δ*Q*). The LV volume trace was obtained by fitting the GLS trace to the peak ESV and EDV, assuming that the LV volume curve mirrors the GLS curve. Mitral flow rate was calculated as the time derivative of the volume trace (dV/dt). This is explained in Supplementary data online, *Data E*. It is referred to *Table 3C* for inputs. Supplementary data online, *Data D* explains details and includes an example case (see Supplementary data online, *Figure S5*) showing how the transmitral pressure difference was calculated.

**Table 3:** Input variables used for calculating (A) the minimum LV pressure, (B) the time constant (τ) of LV pressure decay, and (C) the maximum atrio-ventricular pressure difference

	Symbol	Description	Units
A	$P_{\mathrm{sys}}$	Systolic aortic pressure	mmHg
	BMI	Body mass index	kg/m^2^
	LARS	LA reservoir strain	%
	τ	Time constant of LV pressure decay	ms
B	$t_{\mathrm{AVC}}$	Time of aortic valve closure	s
	$t_{\mathrm{MVO}}$	Time of mitral valve opening	s
	$P_{\mathrm{MVO}}$	P_LV_ at mitral valve opening	mmHg
	$P_{\mathrm{sys}}$	Systolic LV pressure	mmHg
C	$v_{\mathrm{MV}}$	Velocity across the mitral valve	m/s
	*Q*	Flow rate trace across mitral valve	mL/s
	ΔQ	Change of flow rate	mL/s

##### Method to estimate the time constant τ of LV pressure decay

The time constant of LV pressure decay during isovolumic relaxation (τ) is defined according to an exponential decay model of diastolic pressure as proposed by Weiss *et al*.^8^:


(4) $$P_{\mathrm{LV}}(t)=\;P_{0}\;*\;e^{-\frac{t}{\tau }}$$

where *e* is the natural logarithmic base, t is time, and P_0_ is LV pressure at time of peak negative LV dP/dt. Our method to estimate τ non-invasively is based on the principles proposed by JD Thomas and co-workers.^11,12^

In the 81 patients of the derivative cohort, we found P_0_ to be 65 ± 7% [mean ± standard deviation (SD)] of peak LV pressure from invasive measurements. For the non-invasive estimate of τ, we assumed P_0_ to be a fixed fraction of 65% of peak LV pressure (P_sys_). Based on observations in previous studies,^7,12–14^ P_MVO_ was assumed to be 5 mmHg higher than mean P_LA_. Thus, P_MVO_ becomes dependent on Eqs. (1) and (2), resulting in a coupled system of equations that is solved by an iterative algorithm (where 10 iterations were enough to obtain a precise convergence of minimum P_LV_, τ, pre-A P_LV_, and P_MVO_). Consistent with the Weiss study,^8^ our estimated time of aortic valve closure (AVC) occurred slightly before peak negative dP/dt. Therefore, we used 85% of isovolumic relaxation time (IVRT) in our τ estimate. For details in measurement of IVRT, it is referred to Supplementary data online, *Data E*.

With these assumptions, τ can be estimated as:


(5) $$\begin{array}{c} \tau =\frac{0.85\cdot \mathrm{IVRT}}{\ln\;(0.65\cdot P_{\mathrm{sys}})-\ln\;(P_{\mathrm{MVO}})} \end{array}$$

The estimation of τ in Eq. (5) depends on the accuracy of the two estimated pressures in the denominator as well as IVRT in the nominator. We investigated the sensitivity of the estimated τ to errors in P_sys_, P_MVO_, and IVRT (see Supplementary data online, *Data C*).

##### Method to estimate end-diastolic P_LV_

Building on the same principles as above, the end-diastolic P_LV_ is computed by adding to pre-A P_LV_ the maximum ΔP_MV_ during the A-wave, and the pressure increase due to passive filling as detailed in Supplementary data online, *Data E*.

##### Validation of pressure estimates

The coefficients in Eqs. (2) and (5) were found from the derivation cohort of 81 patients using invasive data. In the 19 patients used as a testing cohort, the estimated values for minimum P_LV_, τ, pre-A P_LV_, and end-diastolic P_LV_ were compared with their invasive reference values.

### Part 2: non-invasive LV diastolic pressure curve

**Figure 3:**
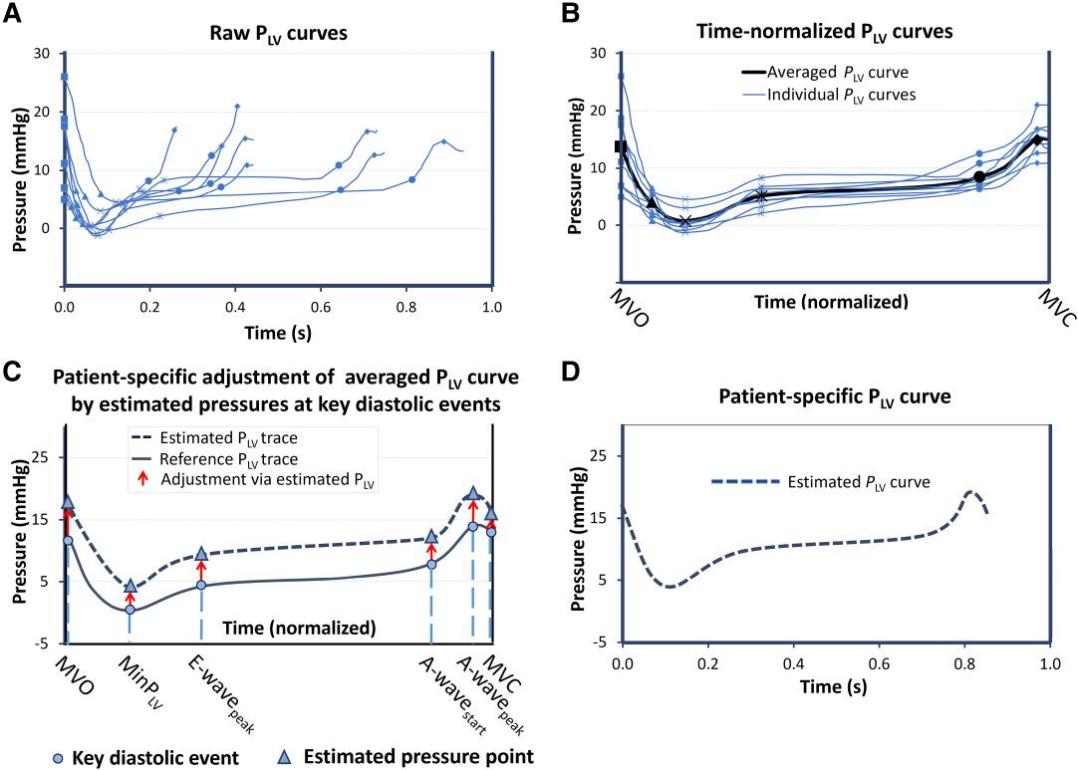
Left ventricular (LV) reference curve and pressure estimates to define patient-specific curves. (*A*) Single patient recordings of LV pressure (P_LV_) from the cohort used to define an averaged diastolic P_LV_ curve. The symbols on the curves indicate key diastolic events. (*B*) Same data as in *A*, showing time-normalized single LV pressure curves and the averaged curve (solid black line). (*C*) Starting with the averaged P_LV_ curve, a patient-specific P_LV_ curve was constructed by shifting pressure at key diastolic events vertically according to the pressure estimate at each event. (*D*) Conversion of the time-normalized P_LV_ curve to a curve with absolute time units involved use of the GLS trace to define the phases of diastole, as illustrated in *Figure 4*.

The second aim of the study was non-invasive generation of a patient-specific LV diastolic pressure curve. As illustrated in *Figure 3A* and *B*, the first step was construction of a time-normalized reference curve with several diastolic events indicated. The second step, which is illustrated in *Figure 3C* and *D*, was to estimate pressure at each diastolic event and to convert the normalized time scale to an absolute time scale. The curve estimates were validated by comparison with invasively measured pressure curves. This approach is somewhat similar to the principle we used in the study by Russell *et al*.^5^ for imaging-based estimation of the LV systolic pressure curve.

#### Reference LV pressure curve

A previously published dataset from Smiseth *et al*.^7^ was used to define a diastolic pressure reference curve. The reference cohort consists of a dataset with eight stable patients referred for evaluation of suspected CAD. Pressures were recorded by micromanometers using dual-sensor 7 F catheters (model no. SSO-654, Millar Instruments, Houston, TX), with one sensor located near the LV base, in the LV outflow tract and one more distally in the ventricle. For construction of the reference pressure curve, we used pressure measured by the distal sensor.

Each individual P_LV_ curve from the reference cohort was first annotated with the occurrence of the key diastolic events: mitral valve opening (MVO), minimum P_LV_, peak E-wave, start and peak A-wave, and mitral valve closure (MVC). Then, the temporal duration between these events was normalized before calculating an averaged pressure curve (panels A and B).

#### Estimated LV pressure curve

The ability to generate patient-specific diastolic LV pressure curves was validated in an independent group of seven patients. They were selected because echocardiography was done while the patients were on the catheterization table that allowed pressures and images to be recorded simultaneously. Echocardiography and LV pressure were recorded from the same series of beats and recordings were done during breath-hold at end-expiration.

**Figure 4:**
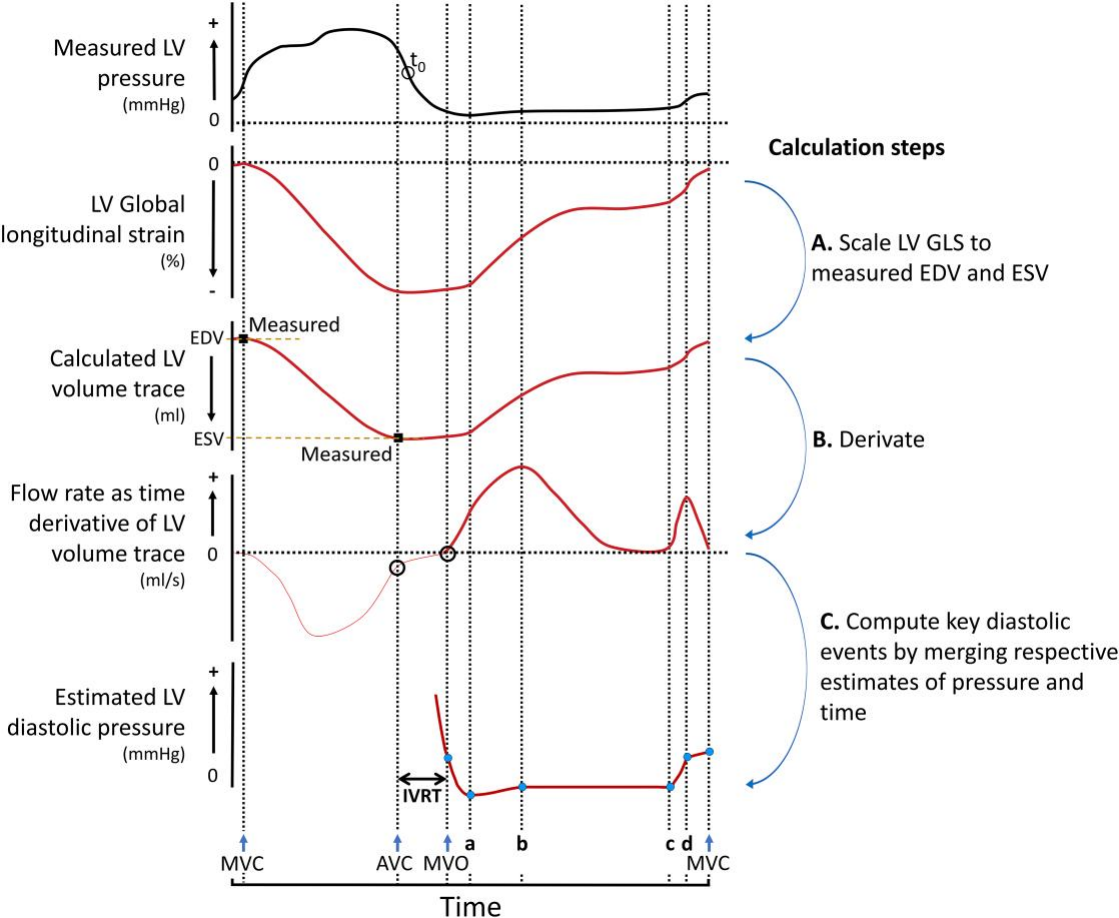
Estimation of isovolumic relaxation time (IVRT) and timing of diastolic events. The LV volume curve and its derivative were used to estimate timing of key diastolic events (cf. *Figure 3* for illustration of time-normalized diastolic pressure curve). The estimated pressure curve was compared with the measured LV pressure curve. t0: start of exponential pressure decay during isovolumic relaxation time (IVRT); AVC, aortic valve closure; MVO, mitral valve opening; MVC, mitral valve closure; a, b, c, and d represent, respectively, minimum P_LV_, peak early-diastolic mitral flow velocity, atrial contraction start, and peak atrial-induced mitral flow velocity.

*Figures 3* and *4* illustrate the principle for estimation of the patient-specific P_LV_ curve by key diastolic events. As illustrated in *Figure 3C*, the value of each pressure point was shifted vertically according to the estimated pressure at each diastolic event. Then, correct timing in the heart cycle of each diastolic event was obtained by using the GLS trace and its derivatives, as illustrated in *Figure 4*. The key diastolic events included the following: MVO, minimum P_LV_, peak mitral E-velocity, pre-A P_LV_, peak mitral A velocity, and MVC. Details of the method to estimate the pressures and timings of these diastolic events are explained in Supplementary data online, *Data E*.

A LV volume trace was inferred by scaling the LV GLS trace to measured end-systolic and end-diastolic volumes, and subsequently computing the corresponding temporal transients of transmitral flow rate [Eq. (6)] and flow acceleration and deceleration [Eq. (7)], as illustrated in *Figure 4*:


(6) $$\begin{array}{c} Q=\frac{\mathrm{dV}}{\mathrm{dt}}\; \end{array}$$

(7) $$\frac{dQ}{\mathrm{dt}}=\frac{d^{2}V}{dt^{2}}$$

where V is LV volume (mL), *Q* is flow rate (mL/s), and t is time (s). Details in calculation of dV/dt are explained in Supplementary data online, *Data E*.

To evaluate this method, the estimated pressure curves were plotted together with the invasively measured curves and compared. Furthermore, the mean pressure from MVO to MVC was computed and compared with the respective measured value.

### Statistical analysis

Values are presented as mean ± SD. Univariate and multivariable linear regression analysis were used to identify predictors of minimum LV pressure and τ.

To evaluate the agreement between estimated and measured LV diastolic pressure key markers and τ, linear correlation and Bland–Altman plots were considered. Correlation based on the linear least squares regression was performed and respective linear equation and *R*^2^ are provided in plots. Bland–Altman plot with 95% limits of agreement was used for comparison between estimated and measured pressures, with the limits of agreement given as 1.96 ∗ SD of the difference. For statistical testing, a value of *P* < 0.05 was considered significant.

## Results

### Estimated LV diastolic pressures

**Figure 5:**
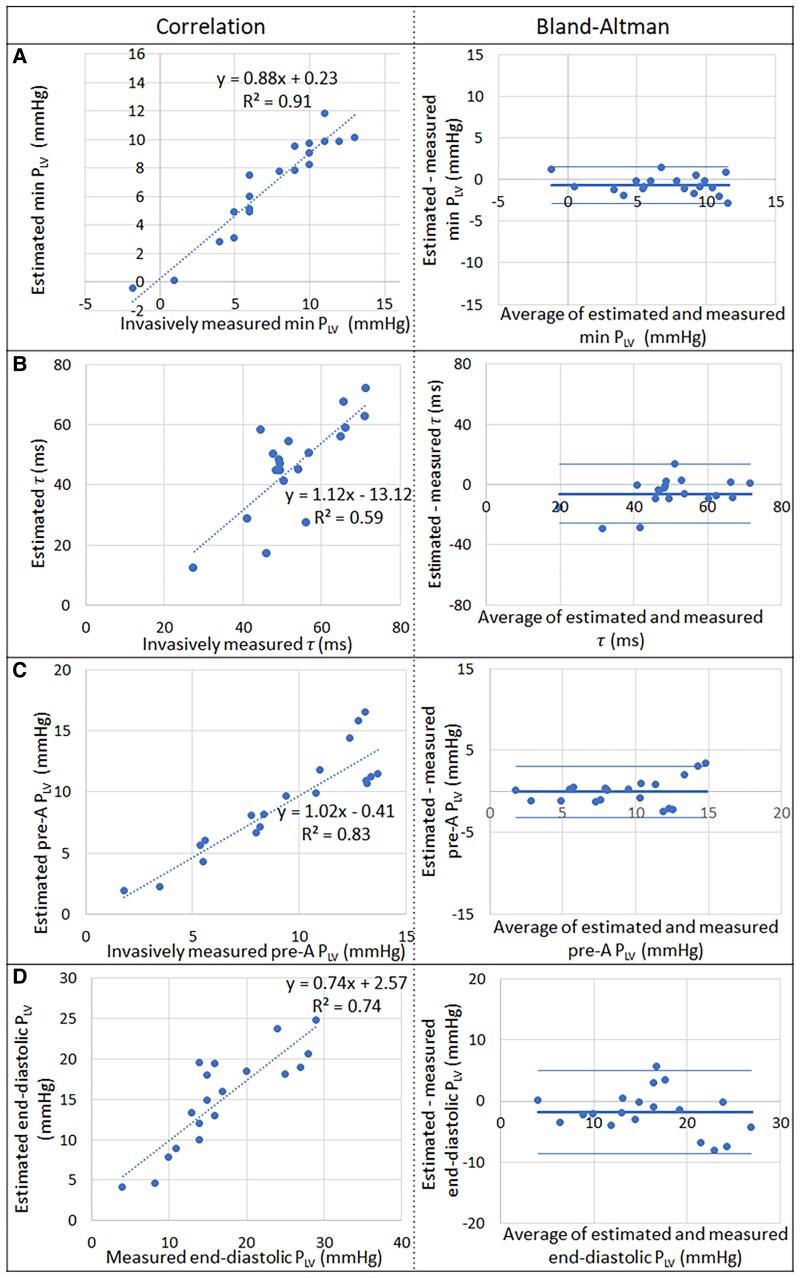
Correlation and agreement between estimated and invasively measured LV diastolic pressures and τ. Correlation and Bland–Altman plots between estimated and invasive measurements in the *n* = 19 patients in the testing cohort: (*A*) minimum left ventricular pressure (min P_LV_); (*B*) isovolumic relaxation time constant τ; (*C*) pre-A P_LV_; (*D*) end-diastolic P_LV_.

*Figure 5* shows agreements between estimated and measured LV diastolic pressures and between estimated and measured τ in the 19 patients in the testing cohort. The estimated minimum P_LV_ had a bias of −0.6 mmHg and limits of agreement < 2.2 mmHg (±1.96 ∗ SD) when compared with invasively measured minimum P_LV_ (*Figure 5A*). The estimated pre-A P_LV_ also had good agreement with its invasive reference value with a bias of 0.0 mmHg and limits of agreement < 3.1 mmHg (*Figure 5C*). The estimates of end-diastolic P_LV_ had a bias of −1.8 mmHg and limits of agreement < 6.8 mmHg when compared with invasively measured end-diastolic P_LV_.

The intermediate variable τ that was required in the estimation of minimum P_LV_ corresponded well with its invasive reference: estimated τ had a bias of −6.8 ms and limits of agreement < 19.5 ms when compared with invasively measured τ (*Figure 5B*). The sensitivity analysis of τ estimation to errors in P_sys_ and P_MVO_ showed that errors of 15 and 5 mmHg, respectively, resulted in errors in τ of 7.3 and 8.6 ms, respectively (see Supplementary data online, *Data C*). For perspective, such can be related to the range of τ from 28 to 71 ms in our population (*Figure 5B*). An error in τ of 8.6 ms will alter the estimated minimum P_LV_ by 1.35 mmHg [via Eq. (2)].

### Estimated LV diastolic pressure curve

**Figure 6:**
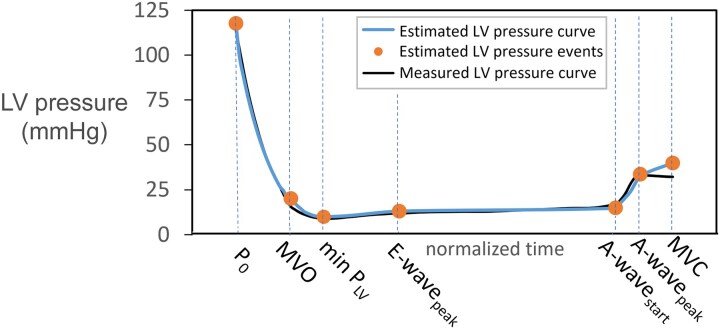
Patient-specific diastolic left ventricular (LV) pressure curve. A representative patient showing a non-invasive estimate of the diastolic LV pressure curve (blue thicker line) and diastolic events (orange dots) in comparison with the measured pressure curve (black thinner line). These diastolic LV pressure events (estimates in orange) include the following: aortic valve closure (AVC), mitral valve opening (MVO), minimum LV pressure (min P_LV_), peak E-wave, end-diastasis (A-wave_start_), peak A-wave (A-wave_peak_), and end-diastole corresponding to mitral valve closure (MVC).

**Figure 7:**
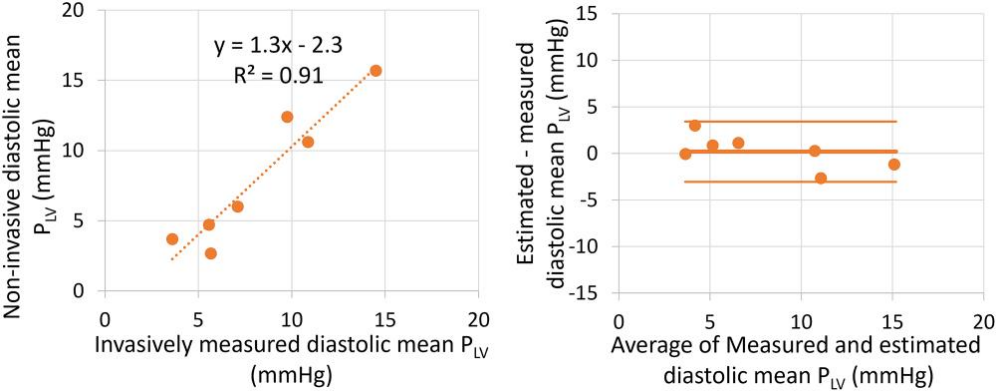
Correlation and agreement between estimated and measured mean diastolic P_LV_ calculated from mitral valve opening to mitral valve closure.

The non-invasive estimate of the diastolic segment of the P_LV_ curve showed good agreement with invasive pressure recordings, both visually and quantitatively by comparing the mean diastolic P_LV_ from t_MVO_ to t_MVC_ (*Figures 6* and *7*). Estimated mean diastolic P_LV_ showed a bias of 0.2 mmHg and limits of agreement < 3.2 mmHg. A sensitivity analysis to the core inputs of the methodology (LV systolic pressure, IVRT, and mitral E-velocity) reported robust results to changes up to 20% (see Supplementary data online, *Data F*).

## Discussion

In the present study, we propose an innovative imaging-based method to estimate LV diastolic pressures non-invasively. The method estimated pre-A P_LV_ as a surrogate for mean P_LA_ with high accuracy when validated against invasively measured pre-A P_LV_. The method also provided estimates of minimum P_LV_, a parameter that reflects LV relaxation and restoring forces, and end-diastolic P_LV_ as measure of preload. Therefore, the method has potential to provide LV functional data that are not accessible with current non-invasive approaches. Additionally, the method offers an estimate of the complete LV diastolic pressure curve, demonstrating good accuracy when validated in a subset of independent patients.

The imaging parameters required for the method are recorded routinely when performing clinical echocardiography, and each parameter has high measurement feasibility. Calculation of the different pressure outputs require data processing that needs to be semiautomated to make the method time efficient as a clinical tool. Since the study included exclusively patients evaluated for CAD and had a relatively small sample size, it should be regarded as a proof-of-principle study for application of the novel method. Ultimately, the utility of the method in clinical practice depends on its accuracy when tested in diverse phenotypes beyond CAD and in larger populations, and such studies are planned.

### Principles behind the method

The proposed method to estimate LV filling pressure used pre-A P_LV_ as a surrogate for mean P_LA_ that is consistent with previous studies and current recommendations.^1,9,10^ In patients with mitral stenosis or severe mitral regurgitation, however, mean P_LA_ may markedly exceed pre-A P_LV_. With these limitations in mind, we consider pre-A P_LV_ to be a valid surrogate for mean P_LA_.

A central assumption in our method is that mean P_LA_ can be estimated as the sum of minimum P_LV_ and maximum ΔP_MV_. The validity of this assumption comes from observations in several experimental studies under a wide range of haemodynamic conditions, including studies in failing hearts.^13,15–17^ The physical rationale for the assumption is that end of diastasis (pre-A) represents an equilibrium pressure point between the LV and LA chambers, and that early filling is driven by the difference in pressure when this equilibrium is not reached yet. Details of this proposed mechanism are presented in Supplementary data online, *Data A*.

Minimum P_LV_ was estimated based on a linear regression model using τ, LA reservoir strain, systolic blood pressure, and BMI as predictors. Among them, and considering the physiological range of each, τ was the largest contributor, and was estimated non-invasively using principles similar to Scalia *et al*.^12^ Our estimate of τ was derived from a formula that incorporated measurement of IVRT from the LV strain signal. This approach enables single-beat analysis, which paves the way for future automation of the method. The conventional approach for measuring IVRT, however, is by measuring LV outflow velocities by continuous wave or pulsed Doppler echocardiography.^1^ Whether the LV strain-based method is comparable to the Doppler methods remains to be studied.

The observation that LA reservoir strain, in addition to τ, is a strong predictor of minimum P_LV_ represents a novel finding in this study. It may reflect dependency of LA reservoir strain on minimum P_LA_, which closely approximates minimum P_LV_, rather than a specific association between LA reservoir strain and minimum P_LV_.^18^ An association between LA reservoir strain and minimum P_LA_ is expected due to the important role of suction for LA filling and reservoir function.^19^ The suction is generated as a combined effect of LA relaxation and LV longitudinal shortening and is reflected in the reduction in P_LA_ in early systole. Furthermore, as shown by Hunderi *et al*.,^20^ the LA pressure–volume curve is curvilinear with shallow slope (high reservoir capacity) at low pressures and steeper slope (low reservoir capacity) at higher pressures. Therefore, when minimum P_LA_ is in the lower range at onset LA filling, LA reservoir capacity is high. We suggest that these mechanisms contribute to the correlation between LA reservoir strain and minimum P_LV_.

The finding that systolic blood pressure was an additional, but weak, independent predictor of minimum P_LV_ is consistent with known effects of afterload on LV early-diastolic pressures.^21^ The association between BMI and minimum P_LV_ may reflect an impact of conditions commonly associated with overweight and obesity.

Finally, note that there was no significant independent contribution to infer minimum P_LV_ from e′ that is often used as marker of relaxation, probably because the impact of relaxation on minimum P_LV_ was reflected in the estimated τ.

The early-diastolic transmitral pressure difference, maximum $\Delta P_{\mathrm{MV}}$, the second summand to estimate mean P_LA_ in Eq. (1), was calculated by a simplification of the Navier–Stokes equation of conservation of mass, i.e. of momentum conservation. As shown by JD Thomas and co-workers, inertial forces represent a substantial fraction of the early-diastolic pressure difference across a normal mitral valve and therefore need to be incorporated when estimating transmitral pressure differences for unrestricted mitral flow.^22–24^ This is explained in more detail in Supplementary data online, *Data D*. In mitral stenosis, however, there is dominance of advective forces (i.e. those to create the spatial acceleration into a narrow filling jet) over inertial forces, which justifies using the simplified Bernoulli’s equation^25^ and its correction^26^ to determine severity of the stenosis.

### Alternative methods to estimate LV filling pressure

Recent innovations in artificial intelligence (AI) make use of ‘black box’ approaches, with promising examples in HF diagnostics based on raw echocardiographic data or electrocardiograms.^27,28^ These approaches provide the likelihood of a given HF diagnostic, but do not provide mechanistic insights or clinical interpretation. On the contrary, our method addresses the need for LV diastolic pressures by a synergetic combination of statistic [a linear regression, Eq. (2)] and mechanistic [for relaxation and transmitral pressure differences, Eqs. (3) and (5)] models, a strength in the use of digital twin technologies.^29^

A number of qualitative or semiquantitative echocardiographic methods for evaluating LV diastolic function and filling pressure are available,^1–3^ but none of them can differentiate between mean LA pressure and LV end-diastolic pressure. Furthermore, they do not provide estimates of minimum LV pressure that is a marker of LV relaxation and restoring forces. The same limitations apply to pulmonary capillary wedge pressure that is used as an indirect measure of mean LA pressure. Therefore, potentially our method may provide new insights into diastolic function beyond just providing an estimate of mean LA pressure.

### Uncontrolled factors in the definition of LV filling pressure

A potential challenge with defining and measuring LV diastolic pressure is the existence of intraventricular pressure differences.^30^ In the patient population used to define the reference curve, which included patients with apparently normal LV systolic function, we used a catheter with two pressure sensors. The average difference between the location with lowest and highest minimum P_LV_ was circa 2 mmHg and peak pressure difference occurred during early rapid filling and with lowest pressure in the LV outflow tract.^7^ This would have a small impact on the measured LV minimum pressure. In that study, there was no measurable intraventricular pressure differences in pre-A pressure,^7^ which is as expected in late diastasis when there are low intraventricular flow velocities. Furthermore, as we have shown previously, intraventricular pressure differences are reduced in ventricles with systolic dysfunction.^31^ Therefore, we do not consider regional differences in minimum P_LV_ to be an important limitation in the present study or in hearts with reduced LV systolic function.

However, variations in the RR intervals may influence degree of filling that is a challenge when comparing methods for measuring diastolic pressure. To minimize this influence in the subgroup used for validation of the diastolic pressure curve, echocardiography and pressure measurements were done simultaneously. In the other patients, echocardiography and pressure measurements were performed no more than two hours apart.

Considering these adverse factors of intracardiac variability (both over space and time) the obtained filling pressure limits of agreement (±1.96 ∗ SD) of <3.1 mmHg is a noteworthy outcome of the present study.

### Defining the LV diastolic pressure curve

The solution presented is inspired by our previously published non-invasive methodology to estimate the LV systolic pressure curve and myocardial work.^5,32^ In the present study, a similar approach was used to define a reference diastolic pressure curve and pressures were estimated at several diastolic events. Furthermore, timing of diastolic events in the heart cycle was defined by using GLS to define IVRT and phases of diastolic filling.

Since the pressure curve validation was done in only a small number of patients, this part of the protocol should be considered just a testing of the principle, which should be explored in future studies. Nonetheless, the present findings suggest that the diastolic pressure curved can be estimated by echocardiography.

### Clinical applications

Elevated LV filling pressure is a diagnostic hallmark of HF, and the novel method could play a role in the diagnostics and management of this large group of patients. The method may also be of value in patients with established HF when considering adjustment of medical therapy to prevent or treat pulmonary vascular congestion.

Furthermore, our method provides an estimate of minimum P_LV_ that is a parameter of LV early-diastolic function and is an interesting variable since it reflects LV relaxation and LV restoring forces (elastic recoil).^33,34^ There is limited knowledge, however, about a potential diagnostic value of minimum P_LV_ since it has not been feasible to measure in clinical diagnostics when using conventional (fluid-filled) catheters due to their poor frequency response causing artefacts in the pressure trace in early diastole when LV pressure changes rapidly.^33,35,36^ Low value of minimum P_LV_ is important for efficient LV filling at rest and even more during exercise when reduction in minimum P_LV_ allows increase in transmitral flow rate in normal hearts with little or no increase in LA pressure.^4^ Therefore, minimum P_LV_ reflects an important feature of diastolic function. Potentially, demonstration of elevated minimum P_LV_ as sign of impaired LV relaxation, may be used to identify diastolic dysfunction in patients with normal LA pressure at rest.

Currently, algorithms that combine several echocardiographic parameters are used to differentiate between normal and elevated LV filling pressure but have limited ability to quantify filling pressure. Furthermore, none of these approaches differentiates between mean LA pressure and LV end-diastolic pressure, which represent two different physiological variables. In heart failure patients, mean LA pressure is an important parameter as it determines pulmonary vascular congestion, whereas LV end-diastolic pressure is a more direct reflector of LV preload, which determines stroke volume. The novel method has potential to differentiate between these two physiological variables.

Clinical implementation of the novel method requires development of software that allows rapid calculation and reporting of the estimated pressures, ideally online during an imaging study or as an off-line analysis tool. Such an automatization of the filling pressure and pressure curve generation will become possible by incorporating machine learning approaches that will increase processing speed and robustness of the input data and hence the results. The echocardiographic variables needed to estimate LV filling pressure with the novel method are part of clinical routine, except for LA strain that is not yet widely implemented.

Furthermore, future studies should also investigate if the proposed method for estimating LV diastolic pressures may be combined with LV volumes to calculate LV diastolic stiffness as a diagnostic parameter when evaluating patients suspected of heart failure or other disorders with LV dysfunction. Moreover, in principle the presented methodology may be adapted for imaging with cardiac magnetic resonance that should be investigated in future studies.

### Study limitations

The study is based on analysis in populations that were included in previous publications from the authors of this study (O.A.S., N.O., and K.W.).^6,7^ We consider the retrospective approach justified due to the uniqueness of the database that includes patients studied by echocardiography and LV pressure using micromanometer-tipped catheters combined with simultaneous pressure via a fluid line that is needed to ensure correct absolute pressure.^33,35^ Whereas micromanometers are excellent for measuring pressure waveforms and pressure amplitudes, they tend to drift, which in some cases may cause several mmHg errors in absolute LV diastolic pressure. In the patients in our database, this was adjusted for by simultaneously recording pressure via a fluid line.

In clinical routine, LV pressure is measured via fluid-filled catheters, which is just fine for pre-A P_LV_ and in most cases for end-diastolic pressure, but do not provide the accuracy needed to measure minimum P_LV_ and τ,^33,35^ which were fundamental parameters for development of our method. For ethical reasons, we preferred utilizing our existing database rather than subjecting new patients to a complex invasive protocol with dual pressure recordings to test the novel concept.

The estimation of the LV volume curve was derived from echocardiographic measurements of end-diastolic and end-systolic volumes in combination with GLS, under the assumption that the LV GLS curve closely mirrors the LV volume curve. However, the relationship between LV volume and GLS is not perfectly linear, which may have introduced some degree of error into the volume estimates. Ideally, LV volume assessments should be performed using high-frame-rate 3D imaging, which was not available in our database. Since imaging quality is both patient and operator dependent, the robustness of the method should be further explored and potentially improved by use of AI and semiautomated measurements.

Whereas our dataset was limited by its size, the variabilities in the estimates of LV filling pressure and minimum P_LV_ were small, supporting the validity of the proposed methodology. The accuracy of the estimated LV end-diastolic pressure, however, tended to be lower than for the other LV pressure parameters. We are currently planning a prospective multicentre study for testing the method in a larger population and in different cardiac phenotypes. Potentially, other predictors of minimum P_LV_ could be considered in specific cardiac diseases. Nevertheless, the variables we used to estimate minimum P_LV_, with τ and LA reservoir strain as the unique determinants, are expected to be disturbed in most cardiac diseases with LV dysfunction. Furthermore, the principles used to estimate ΔP_MV_ by Navier–Stokes equation are expected to be valid regardless of phenotype.

## Conclusions

In the present study, we introduce a novel method based on cardiac imaging and fluid mechanics analysis for estimation of LV diastolic pressures. The method estimated LV filling pressure measured as pre-A P_LV_ with good accuracy and provided an accurate estimate of minimum P_LV_ that is a marker of LV relaxation and restoring forces. The method also provided an approximation of the LV diastolic pressure curve. Furthermore, the method has potential to differentiate between LA mean pressure, which is closely related to pulmonary vascular congestion, and LV end-diastolic pressure that determines preload and stroke volume. To determine the clinical utility of the method, validation in larger populations with different phenotypes is needed and is being prepared.

## Supplementary Material

jeaf017_Supplementary_Data

## Data Availability

The data that support the findings of this study are available from the corresponding author upon reasonable request.
