# Correction to: Imaging-based method to quantify left ventricular diastolic pressures

**DOI:** 10.1093/ehjci/jeaf242

**Published:** 2025-08-26

This is a correction to: re Otto A Smiseth, Joao F Fernandes, Nobuyuki Ohte, Kazuaki Wakami, Erwan Donal, Espen W Remme, Pablo Lamata, Imaging-based method to quantify left ventricular diastolic pressures, *European Heart Journal - Cardiovascular Imaging*, Volume 26, Issue 7, July 2025, Pages 1184–1194, https://doi.org/10.1093/ehjci/jeaf017

With the originally-published version of the manuscript, the linked supplementary data file was corrupted and could not be opened. The publisher apologises for all inconvenience and delay in having a new file republished with the article.

