# Supplementary material for: Imaging-based method to quantify left ventricular diastolic pressures
Source: Eur Heart J Cardiovasc Imaging. 2025 Jan 16;26(7):1184–94. doi: 10.1093/ehjci/jeaf017 (PMC12206579; doi:10.1093/ehjci/jeaf017)
Supplement: jeaf017_Supplementary_Data [file jeaf017_supplementary_data.docx]

# Supplementary Data

## Supplementary Data A: Estimation of LV pre-A pressure

The method assumes that LV pre-atrial contraction pressure (pre-A P_LV_) approximates mean pressure in the left atrium (P_LA_), which is supported by previous clinical studies ^(9, 10)^. Furthermore, as shown in several experimental studies in conscious and anaesthetized dogs under a wide range of haemodynamic conditions including congestive heart failure, mean P_LA_ can be approximated as the sum of minimum P_LV_ and maximum early-diastolic transmitral pressure difference (∆P_MV_) ^(11-13)^. These historical data are summarized in Supplemental Figure S1, panels B and C.

Based on these concepts, pre-A P_LV_ was calculated according to the following equation:

$$\text{LV filling pressure}=\text{pre-A}P_{\mathrm{LV}}{\approx minP}_{\mathrm{LV}}+{max\Delta P}_{\mathrm{MV}} \left( Eq.1 \right)$$

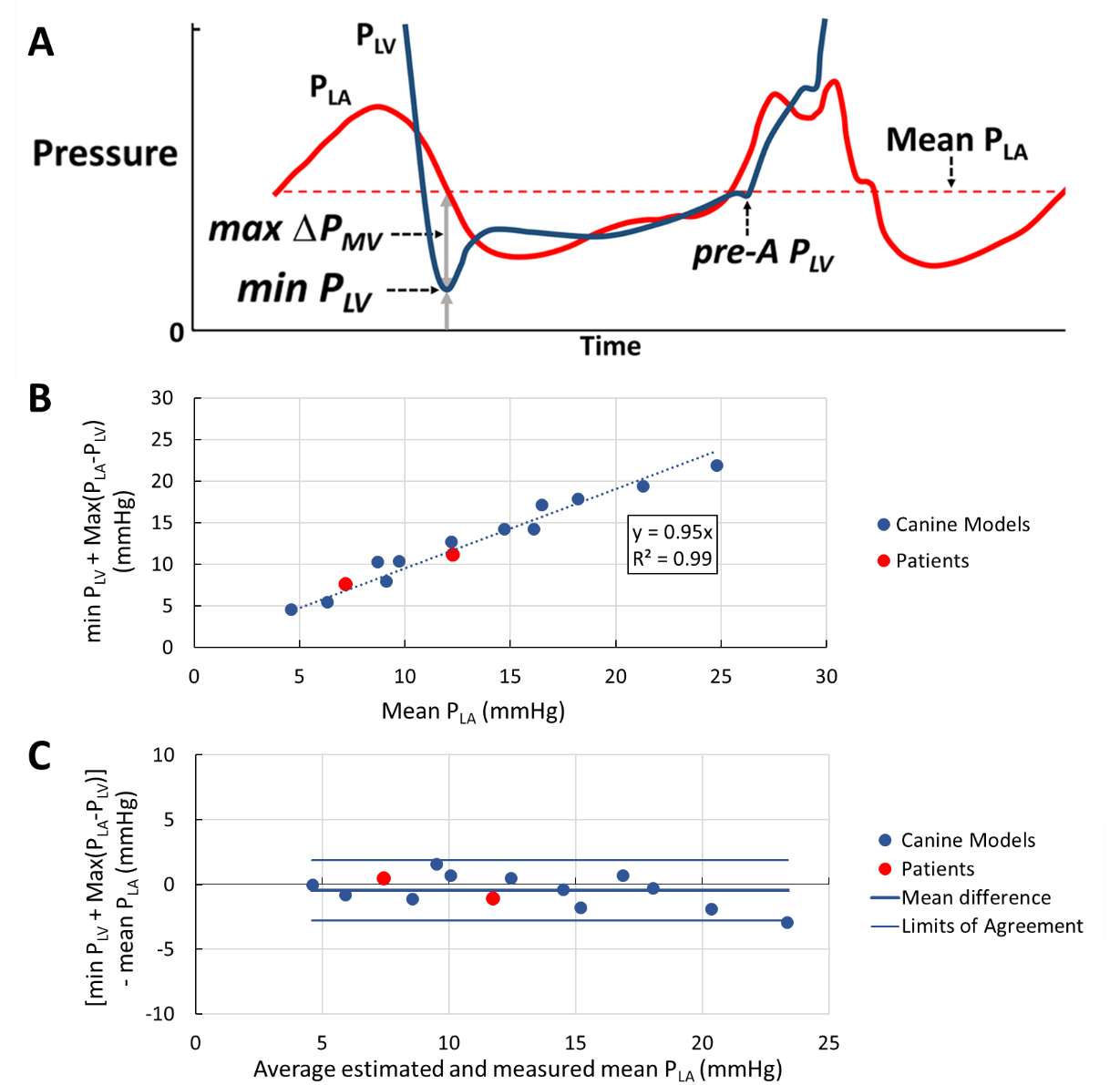


Figure S1: **Mean P_LA_ defined from pre-A P_LV_** Panel A: Schematic illustration of left atrial (LA) and left ventricular (LV) diastolic pressures. Pressure parameters used in the analysis are indicated. Panels B and C: Correlation and agreement between invasively measured mean LA pressure (P_LA_) and the sum of the peak early-diastolic transmitral pressure difference (∆P_MV_ = P_LA_ - P_LV_) and minimum P_LV_ (min P_LV_). The blue dots represent mean data from studies by Hasegawa et al. in conscious dogs with HF ^(11)^, by Ishida et al. in conscious dogs during increased afterload by vasoconstrictor drugs and elevated preload ^(12)^, and by Yamamoto et al. in anaesthetized dogs during different loading conditions and during acute HF ^(13)^. The two red dots are recordings from two different patients (from study by Smiseth et al ^(32)^).

## Supplementary Data B: Construction of the model that infers minimum LV pressure

### Introduction

The minimum P_LV_ is one of the two summands to estimate filling pressure (see equation 1 in manuscript) and is one of the key diastolic events (i.e., pressure-time points) of the diastolic P_LV_ curve. The value of minimum P_LV_ is estimated by a statistical model, by an inference of a multivariable regression model. This supplementary section details the process for the selection of variables and training of this model.

### Data

The model to infer minimum P_LV_ was trained on the cohort from Yoshida et al, 2006 that included patients with suspected CAD ^(6)^. High fidelity micromanometer-tipped catheters were used (Millar Instruments, Houston, Texas, US) to capture minimum P_LV_. The total cohort of 100 patients were divided into the derivation cohort of 81 patients, and the test cohort with the 19 patients which also included LV strain recordings as part of the echocardiographic protocol.

### Methodology

A model to infer the measured minimum P_LV_ was built using standard markers of diastolic function as predictors. A multivariable linear regression model was chosen for its simplicity, and 10 variables related to diastolic performance were selected as potential predictors: indexed LV end-systolic volume (ESVi), indexed left atrial maximum volume (LAVi), ratio of peak mitral E to A velocity (E/A), ratio of peak mitral E velocity to peak longitudinal mitral tissue velocity (E/e’), peak systolic arterial pressure (P_sys_), body mass index (BMI), LA reservoir strain (LARS), LA pump strain (LAPS), peak longitudinal mitral tissue velocity (e’) and time constant (*τ*) of LV isovolumetric relaxation. A first complete regression model was built with all 10 variables, and then a gradual reduction of the model was performed until all remaining variables were statistically significant predictors.

The importance of each of the final predictors was assessed by the range of minimum P_LV_ that was predicted by the range of variation of each predictor (i.e., by the product of the linear coefficient of the regression and the standard deviation of the predictor variable). The standard deviation in each input predictor was assessed in our test cohort (i.e., the 19 subjects). We finally explored the potential limits of generality of our model by comparing the range of variation of the predictors of minimum P_LV_ in the general population (as found in the literature).

### Results

The initial full model in the inference of minimum P_LV_ with n =10 diastolic variables had a regression error of 0.0 ± 2.0 mmHg and found 3 statistically significant variables (Table S1). The final reduced model found four significant predictors (Table S2) and reported a very good performance similar to the initial model, with a regression error of 0.0 ± 2.1 mmHg.

The most important of these four predictors were *τ*, followed by LARS, P_sys_ and BMI (see Table S3). Our population of CAD subjects did show a narrower range of BMI values compared to that of the general population, (Table S3, a vs. b). Furthermore, we calculated the potential residual errors on minimum P_LV_ estimation due to measurement errors of each predictor previously reported in literature or present in our data ^(33-35)^, and found that errors in τ induce the bigger error in minimum P_LV_, but still within a reasonable range comparing to invasive minimum P_LV_ measurements (Table S4).

**Conclusion**

The linear model of independent parameters that best inferred minimum P_LV_ included the four variables, ranked by importance: *τ*, LARS, P_sys_ and BMI. The test cohort did not have patients with BMI >30 kg/m2, so the behaviour of this model has not been evaluated in obese subjects.

Supplemental Table S1: Initial multivariable linear regression model with 10 variables for estimation of minimum $P_{LV}$ in the 81 patients of the derivation cohort. Coef: coefficient; std: standard deviation, err(t): error term; P>|t|: p-value; [0.025 0.975]: limits of agreement; ESVi: indexed left ventricular end-systolic volume; LAVi: left atrial indexed volume; E/A: Ratio of E to A velocity; E/e’ : ratio of peak E velocity to peak mitral tissue velocity; P_sys_: peak systolic pressure; BMI: body mass indexed; LARS: left atrial reservoir strain; LAPS: left atrial pump strain; e’: peak mitral tissue velocity; τ: isovolumetric relaxation constant.

|  | Coefficient | std | Err (t) | P>\|t\| | [0.025 | 0.975] |
| --- | --- | --- | --- | --- | --- | --- |
| **constant** | -8.7083 | 4.509 | -1.931 | 0.057 | -17.700 | 0.284 |
| **ESVi** | 0.0218 | 0.012 | 1.802 | 0.076 | -0.002 | 0.046 |
| **LAVi** | -0.0102 | 0.030 | -0.337 | 0.737 | -0.071 | 0.05 |
| **E/A** | 0.1397 | 0.761 | 0.184 | 0.855 | -1.378 | 1.658 |
| **E/e’** | -0.0546 | 0.124 | -0.439 | 0.662 | -0.303 | 0.193 |
| **P_sys_** | 0.0306 | 0.013 | 2.432 | 0.018* | 0.006 | 0.056 |
| **BMI** | 0.2659 | 0.083 | 3.196 | 0.002* | 0.100 | 0.432 |
| **LARS** | -0.0607 | 0.045 | -1.346 | 0.183 | -0.151 | 0.029 |
| **LAPS** | -0.0481 | 0.062 | -0.777 | 0.440 | -0.172 | 0.075 |
| **e’** | 0.0857 | 0.241 | 0.355 | 0.723 | -0.395 | 0.567 |
| **τ** | 0.1456 | 0.037 | 3.955 | 0.000* | 0.072 | 0.219 |

Supplemental Table S2: Final reduced multivariable linear regression model with four independent variables for estimation of minimum P_LV_ in the 81 patients of the derivation cohort. Std: standard deviation, err(t): error term; P>|t|: p-value; [0.025 0.975]: limits of agreement; P_sys_: peak systolic pressure, BMI: body mass indexed; LARS: left atrium reservoir strain (LARS), left atrium pump strain (LAPS), τ: isovolumetric relaxation constant τ.

|  | coefficient | std | Err(t) | P>\|t\| | [0.025 | 0.975] |
| --- | --- | --- | --- | --- | --- | --- |
| **constant** | -6.8853 | 3.499 | -1.968 | 0.053 | -13.854 | 0.084 |
| **P_sys_** | 0.0250 | 0.012 | 2.165 | 0.034* | 0.002 | 0.048 |
| **BMI** | 0.2430 | 0.079 | 3.061 | 0.003* | 0.085 | 0.401 |
| **LARS** | -0.0973 | 0.026 | -3.684 | 0.000* | -0.150 | -0.045 |
| **τ** | 0.1575 | 0.031 | 5.018 | 0.000* | 0.095 | 0.22 |

Supplemental Table S3: Predictors ranked by importance, by the range of change in minimum P_LV_ inferred by each of them from the linear regression model. The range of variation of input variables is estimated from the test cohort (a) and from literature values of the general population (b).

| **Predictor** | **a) In test cohort**  Mean ± SD of predictor | Change of $minP_{LV}$ | **b) In general population**  Range of predictor | Change of $minP_{LV}$ |
| --- | --- | --- | --- | --- |
| **τ** | 46.5 ± 15.5 ms | + 7.3 ± **2.4** mmHg | 25 – 75 ms ^(36, 37)^ | 3.9 – 11.8 mmHg |
| **LARS** | 31.2 ± 11.7 % | - 3.0 ± **1.1** mmHg | 5 – 55 % ^(33)^ | -0.5 – -5.4 mmHg |
| **P_sys_** | 146.3 ± 22.8 mmHg | + 3.7 ± **0.6** mmHg | 90 – 180 mmHg ^(34)^ | 2.3 – 4.5 mmHg |
| **BMI** | 23.4 ± 1.9 kg/m^2^ | + 5.7 ± **0.5** mmHg | 17 – 35 kg/m^2^ ^(35)^ | 4.1 – 8.5 mmHg |

Supplemental Table S4: Expected range of measurement error in predictors (as present in our data or previously reported in literature) and respective error margin in minimum P_LV_ estimation corresponding to each input error range.

| **Predictor** | Measurement error | Error in $minP_{LV}$ |
| --- | --- | --- |
| **τ** | ± 9.9 ms | ± 1.6 mmHg |
| **LARS** | ± 5.4 % ^(33)^ | ± 0.5 mmHg |
| **P_sys_** | ± 5 mmHg ^(34)^ | ± 0.1 mmHg |
| **BMI** | ± 0.4 kg/m2 ^(35)^ | ± 0.1 mmHg |

## Supplementary Data C: Sensitivity to assumptions in the estimation of *τ*

### Introduction

The LV isovolumetric relaxation constant, *τ*, is one of the four variables used in the estimation of minimum P_LV_. Based on previous works ^(14, 15, 37)^, a non-invasive estimation of *τ* is proposed based on the assessment of the IVRT and two pressure estimates, one at the maximum pressure decay during isovolumetric relaxation (P_0_) and another at a time slightly before of mitral valve opening, P_MVO_ (corresponding to 5 mmHg higher than P_MVO_). This supplementary section includes a sensitivity analysis to errors in these three variables.

### Methods

The sensitivity to input errors around a reference estimate of *τ* using equation 5 taking baseline values of IVRT = 87.1 ms, P_0_ = 86.7 mmHg, and P_MVO_ = 21 mmHg was computed. The sensitivity of *τ* to errors in each of these three parameters was found by calculating the change in *τ* for a variation of ± 5 ms in IVRT, ±10 mmHg in P_0_, and ± 5 mmHg in P_MVO_. These ranges are considered as a realistic error considering human physiology and current technological capabilities.

### Results

Figure S2 shows the variation in *τ* for a range of errors in each of the three input parameters. An error of ± 5 ms in IVRT translated into an error of ± 3.9 ms in *τ*. An error of ± 10 mmHg in P_0_, which corresponds to error of ±15 mmHg in P_sys_ (*P_0_ ≈ 0.65 * P_sys_*), translated into an error of ± 7.3 ms in *τ*. An error of ± 5 mmHg in MVO P_LV_ translated into an error of ± 8.6 ms in *τ*. In context, an error of ± 5 ms in *τ* caused an error of ± 0.8 mmHg in minimum P_LV_ according to equation 2.

### Conclusion

The estimation of *τ* is most sensitive to errors in MVO P_LV_. Furthermore, and as studied by Myreng and Smiseth, under increasing preload, the association between IVRT and *τ* might be altered ^(37)^.


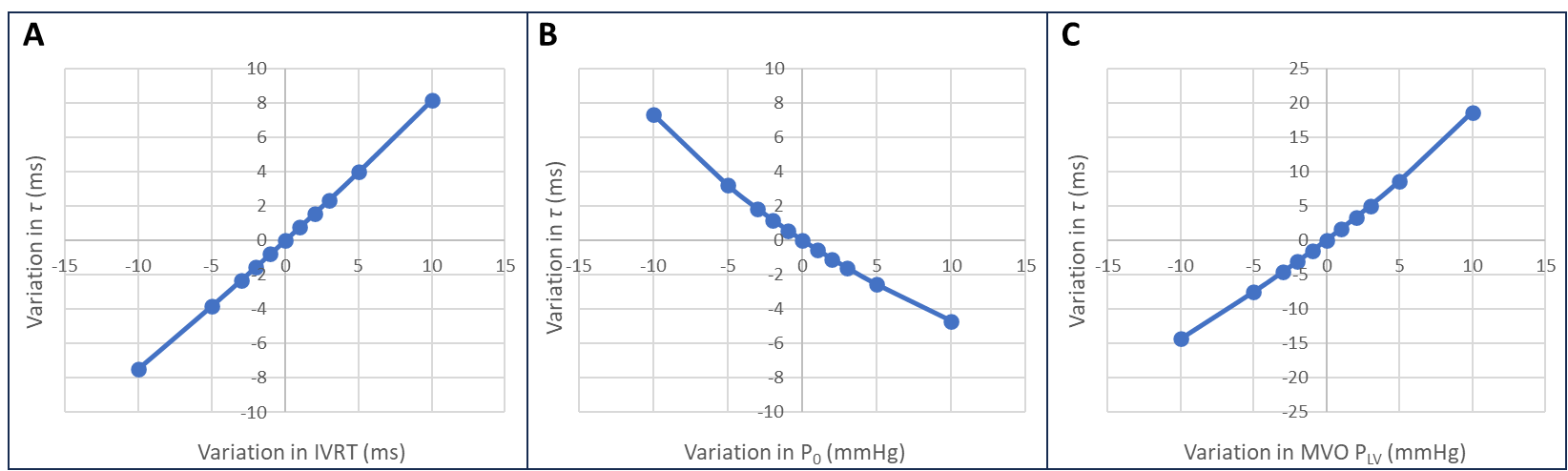
 Supplemental Figure S2: Sensitivity of the estimated τ to variations in the three parameters used in its estimation: A) Isovolumetric relaxation time (IVRT), B) pressure at start of exponential pressure decay during isovolumetric relaxation (P_0_), and C) LV pressure at mitral valve opening (P_MVO_).

## Supplementary Data D: Calculation of maximum transmitral pressure drop

### Introduction

The maximum atrio-ventricular (i.e., transmitral) pressure drop (∆P_MV_), during early filling, is one of the two summands to estimate LV pre-A pressure (equation 1 in manuscript). This supplementary section explains the simplification of the Navier-Stokes equation that is used to estimate maximum ∆P_MV_. Furthermore, the same principles are applied to late filling pressure drop, which is used in the estimation of end-diastolic LV pressure.

### The Navier-Stokes equation and assumptions

The Navier-Stokes momentum conservation equation is the application of Newton’s second fundamental law of physics (i.e., force equals mass times acceleration) to flow. Pressure is force divided by the area, and the pressure difference ΔP between two points along a vessel accounts for three summands ^(38, 39)^, blood acceleration in time (i.e. temporal acceleration of kinetic energy, ΔP_K_, also called inertial term), blood acceleration in space (i.e. convective summand, ΔP_C_, also called advective term) and dissipation by friction (i.e. viscous summand, ΔP_V_):

$$\begin{aligned} \Delta P=\Delta P_{\text{K}}+\Delta P_{\text{C}}+\Delta P_{\text{V}} =-\frac{1}{Q}\left( \frac{\partial K}{\partial t}+C+V \right)\#\left( eq. S1 \right) \end{aligned}$$

where Q is the flow rate, ∂K⁄∂t is the temporal derivative of the kinetic energy (i.e. temporal acceleration term), C is the convective energy rate (also mentioned as advective, and which characterizes the balance of flow momentum in and out), and $V$ is the rate of viscous dissipation describing energy losses because of friction. Equation S1 has been derived by the Work-Energy Relative Pressure formulation ^(21, 39)^. Note as well that pressure drop is considered to be positive, as the pressure in the LV is lower than in the LA, and the LV is the focus here.

The simplification taken for the computation of maximum ∆P_MV_ is based on these assumptions:

1. The viscous term, ΔP_V_, is neglected because of its very small magnitude compared to the other two terms when flow crosses cardiac valves ^(19, 21)^.
2. The convective term is approximated using the simplified Bernoulli equation ^(20)^, that requires the maximum value of velocity across the valve at a given time instant:

$$\begin{aligned} \Delta P_{\text{C}}= {\frac{\rho}{2}.v}_{\text{Max}}^{2}\approx4 v_{\text{Max}}^{2}\#\left( eq. S2 \right) \end{aligned}$$

As such, the convective term peaks at the instant of peak velocity across the valve when the local temporal acceleration is null ^(38)^, see Figure S3.

1. The temporal acceleration term (∆P_K_) is computed as the pressure variation due to the temporal change of blood flow inertia, i.e. by acceleration over time of the volume of blood crossing the MV ^(19)^, and its derivation is explained in next section. The temporal acceleration term does not peak at the instant of peak velocity, but before while blood is being accelerated ^(38)^, see Figure S3.
2. The instant when maximum ∆P_MV_ occurs depends on the relative contribution of the convective and temporal acceleration summands. The search for this instant is restricted within the temporal interval between these two events: from instant of peak acceleration to the instant of peak velocity (peak convective term). Such is valid for both E- and A-waves, see Figure S3.


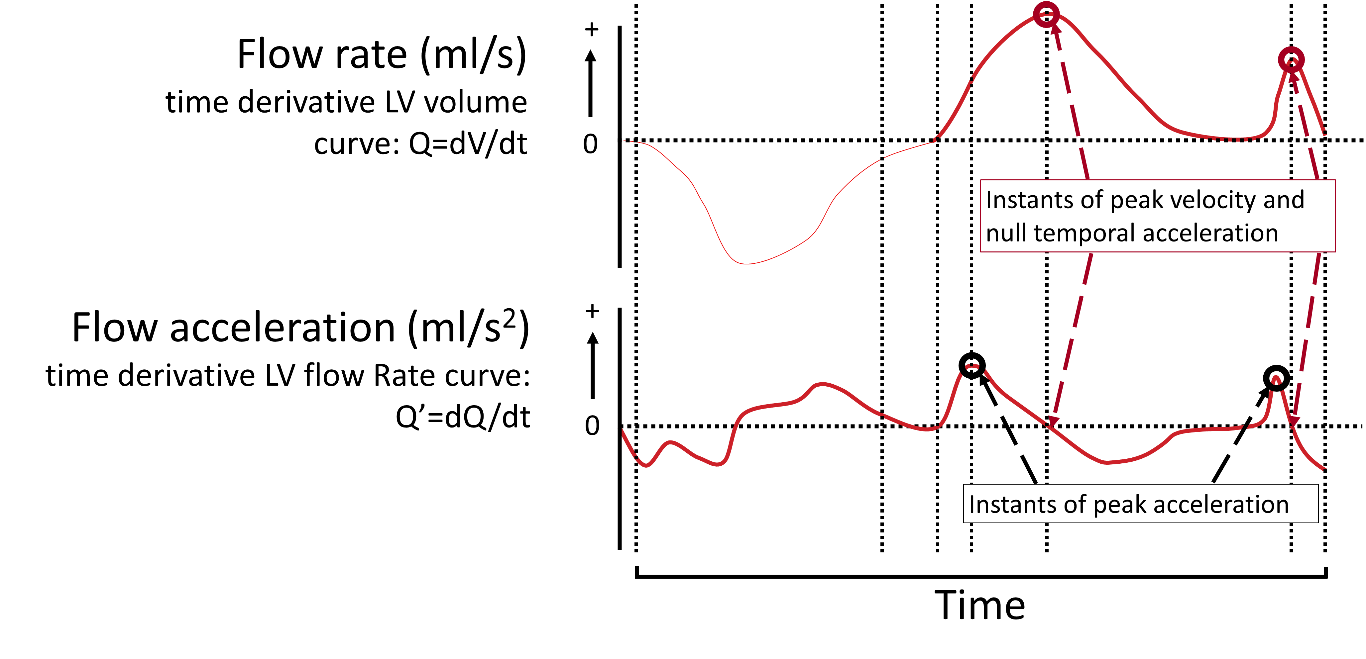


Supplemental Figure S3: Schematic of the two key time instants for the estimation of the transmitral (atrio-ventricular) pressure drop during E-wave and A-wave: the instant of peak flow velocity, i.e. when the convective term is maximum, and the instant of peak acceleration, i.e. when the kinetic term is maximum.

### Derivation of the kinetic term

The pressure drop caused by the kinetic term is computed by the temporal change of momentum of a blood particle:

$$\begin{aligned} {dP}_{K}=-\frac{1}{Q}\left( \frac{\partial K}{\partial t} \right)=-\frac{1}{Q}\frac{\partial}{\partial t}\left( \frac{\text{m}v^{2}}{2} \right)\#\left( eq. S3 \right) \end{aligned}$$

where Q is a flow rate, m is mass of blood, $v^{2}$ is the squared value of velocity, t is the time instant and ∂ is the differentiation.

The complete computation of the kinetic energy term would require an integration across the full volume of flow that is being accelerated or decelerated, i.e., the entire flow domain of atrium and ventricle. Such integral would require either the availability of a dense velocity field sampled across the full atrio-ventricular domain and across time, or complex physics and modelling applied in a range of structures from the pulmonary veins until the LV apex. Four approximations are taken to circumvent this need:

1. A single velocity value, the peak velocity across the mitral valve at a given instant, is assumed across all the domain of computation, neglecting its spatial variations. The instant considered is the one of peak temporal acceleration that is when the kinetic term is maximum ^(38)^, $v_{\mathrm{PeakAcc}}$.
2. The initial momentum is assumed to be null. Temporal acceleration is computed not by continuous derivatives, but by discrete finite differences during the acceleration interval $\Delta t$ that is required to accelerate from null to the momentum at a given instant.
3. The domain of integration is simplified to the filling jet across the mitral valve, since this is where the biggest change of momentum occurs. This domain of integration is assumed to be a column of blood that has entered the ventricle during the acceleration interval ∆t.
4. The size of the column of blood, i.e. the amount of mass of blood being accelerated, can be estimated as the product of the blood flow density (ρ = 1060 kg/m3) and the blood volume ∆V that crossed the valve during the considered acceleration interval ∆t, The instantaneous differential increment is dV=Q∙dt, and an heuristic approximation of its temporal integral is taken as ∆V=Q∙dt∙Q/∆Q, where ∆Q is the flow increment in time (i.e. difference of flow rates between two specific consecutive frames, with an exemplary temporal resampling of the heart cycle of 72 points).

With these assumptions, we obtain the approximation only valid at the instant of peak acceleration:

$$\begin{aligned} {\Delta P}_{K}=\frac{\text{ρ}\text{Q} v_{\mathrm{PeakAcc}}^{2}}{2 \Delta Q}\#\left( eq. S4 \right) \end{aligned}$$

Eventually, this final expression allows the estimation of the peak kinetic pressure drop across the mitral valve for given E-wave and A-wave peak acceleration time points.

### Conclusion

The estimate for maximum ∆P_MV_ occurring during E-wave (Figure S3) is computed as the maximum across the events of maximum velocity or maximum acceleration:

$$\begin{aligned} max{\Delta P}_{\mathrm{MV}}(t)\approx max\left( \frac{\rho}{2}\left( {v(t)}_{\text{MV}}^{2}+\frac{Q\left( t \right). {v(t)}_{\text{MV}}^{2}}{\Delta Q(t)} \right) \right),\#\left( eq. S5 \right) \end{aligned}$$

using the traces of transmitral velocity (v_MV_), the flow rate (Q), and its increment in time (ΔQ). The same principles can be used to estimated the maximum ${\Delta P}_{\mathrm{MV}}$ during A-wave.

## Supplementary Data E: Calculation of diastolic markers

In order to generate non-invasively each patient-specific diastolic P_LV_ curve, a set of key diastolic events (i.e. pressure-time points in the curve) are needed. This supplementary section explains the estimation of the values of pressure and time of these events.

Supplemental Table S5: Key diastolic events used for the LV pressure curve construction.

| Symbols | Description | Units |
| --- | --- | --- |
| $\left( P_{\text{0}},t_{\text{0}} \right)$ | P_LV_ and time at minimum dP/dt | mmHg, seconds |
| $\left( P_{\text{MVO}},t_{\text{MVO}} \right)$ | Mitral valve opening | mmHg, seconds |
| $\left( P_{\text{min}},t_{\text{min}} \right)$ | Minimum LV pressure | mmHg, seconds |
| $\left( P_{\text{E-peak}},t_{\text{E-peak}} \right)$ | Peak of mitral E-wave velocity | mmHg, seconds |
| $\left( P_{\text{A-start}},t_{\text{A-start}} \right)$ | Start of A-wave velocity | mmHg, seconds |
| $\left( P_{\text{A-peak}},t_{\text{A-peak}} \right)$ | Peak of A-wave velocity | mmHg, seconds |
| ${(P}_{\mathrm{MVC}},t_{\mathrm{MVC}})$ | Mitral valve closure (end diastole) | mmHg, seconds |

Therefore, the objective is the estimation of the key diastolic events (14 variables, i.e. 7 time instants and 7 pressure values) described in Table S5. The estimation of time instants makes use of the temporal transients of LV volume. Given that, in current clinical practise LV volume traces are not available, but, instead global longitudinal strain (GLS) traces are, the LV volume trace was inferred by scaling the LV GLS trace to measured end-systolic and end-diastolic volumes, and subsequently computing the corresponding temporal transients of filling flow rate (equation 6, *Q = dV/dt*) and flow acceleration and deceleration (equation 7, *dQ/dt = d^2^V/dt^2^*), as illustrated in Figure 4 in main manuscript.

The detailed description of how to calculate each key diastolic event is provided next.

1. **Minimum dP/dt (P_0_, t_0_)**, see Figure S4. As explained in the methods subsection of *τ* estimation in the manuscript, P_0_ was estimated as *P_0_ ≈ 0.65⋅P**_sys_* based on the derivation cohort of 81 patients from Yoshida et al. ^(6)^, where P_sys_ is an estimation of peak systolic pressure in the LV in cases without aortic stenosis. Consistently with the works by Weiss et al.^(8)^, our estimated time of AVC occurred slightly before peak negative dP/dt (P_0_). Thus, and in order to reassure that only an exponential behaviour of the isovolumetric pressure decay was considered, we used 85 % of isovolumetric relaxation time (IVRT) for *τ* estimation. The IVRT was estimated from AVC to MVO, where AVC is defined as time at peak positive second derivative of flow immediately before isovolumetric relaxation, i.e. the end of flow deceleration into aorta before the period of stable minimal volume, as introduced by Remme et al. ^(40)^.

**
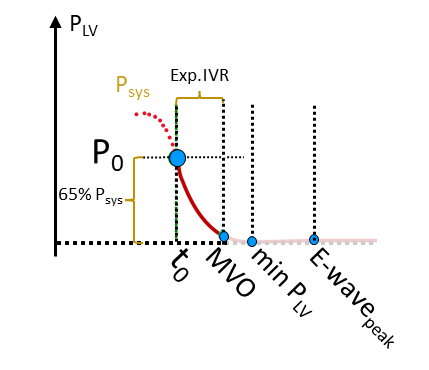
**

**Supplemental Figure S4:** Schematic of the estimation of pressure-time point of maximal pressure decay (P_0_) during isovolumetric relaxation. Exp. IVR: Exponential section of the pressure decay during isovolumetric relaxation; t_0_: instant when P_0_ occurred.

1. **Mitral valve opening (P_MVO_, t_MVO_)**, see Figure S5: As explained in the computation of *τ* in the manuscript, *P_MVO_ = (meanP_LA_ + 5 mmHg)* ∙ *t_MVO_*, t_MVO_ is the first time point after t_AVC_ when transmitral d*V*/d*t* and d*Q*/d*t* are positive.


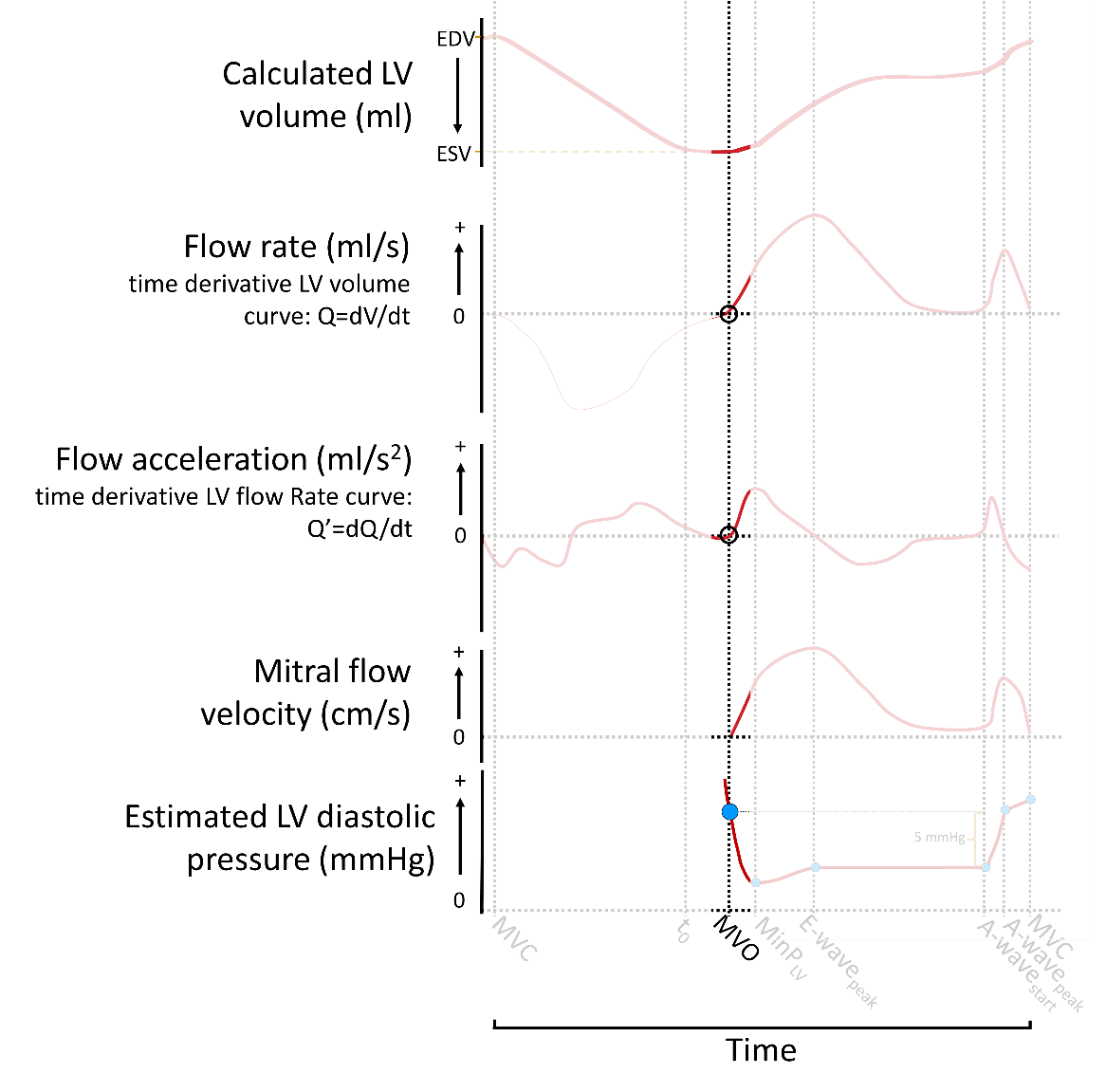


**Supplemental Figure S5:** Schematic of the estimation of pressure-time point of mitral valve opening (MVO) with timing based on onset of flow rate and flow acceleration traces (open circles).

1. **Minimum LV pressure (P_min_, t_min_)**, see Figure S6: The estimate of P_min_ is given in equation 2 in the main manuscript, and occurs about the time of maximum LA-to-LV pressure difference during early filling, hence t_min_ is the time point of peak d*Q*/d*t* (i.e. peak acceleration) within the range of 45-80 % of the time interval between t_MVO_ and t_E-wave_. The time range was determined by combining data collected from previous studies ^(7, 41, 42)^.


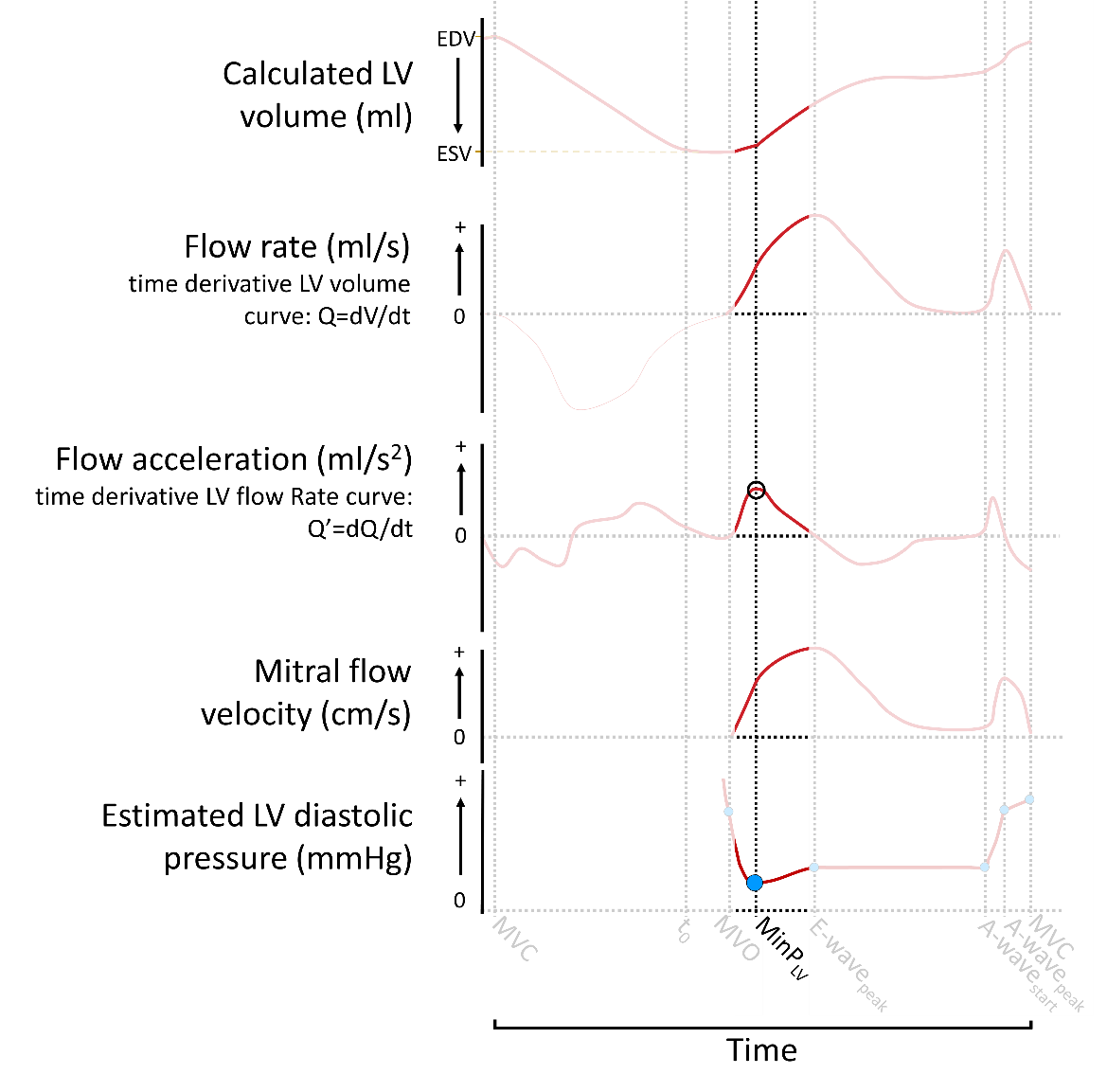


**Supplemental Figure S6:** Schematic of the estimation of pressure-time point of minimum LV pressure (min P_LV_), with timing based on peak flow acceleration (open circle).

1. **Peak E-wave (P_E-wave_, t_E-wave_)**, see Figure S7: The estimate of P_E-wave_ is based on the end-diastolic pressure-volume relationship, assumed to be linear during diastasis from E-wave peak velocity until the start of A-wave as described by Zile et al. ^(43)^. As such, the LV volume (V) is converted to pressure through a linear equation *P_E-wave_ ≈ aV + b*, where stiffness constants (a, b) are computed per each patient based on two pressure-volume points: the first is the hypothetical fully relaxed LV configuration defined by Weiss et al, 1976 ^(8)^ (i.e. pressure theoretically following isovolumetric relaxation exponential decay dictated by *τ*, as defined in equation 4, at instant of minimum P_LV_), and on the estimated pressure and volumes at the start of A-wave. Exemplary values are *a = 0.2*, *b = -8.1* ∙ *t_E-wave_* is the instant of peak flow rate, when d*Q*/d*t* = 0.


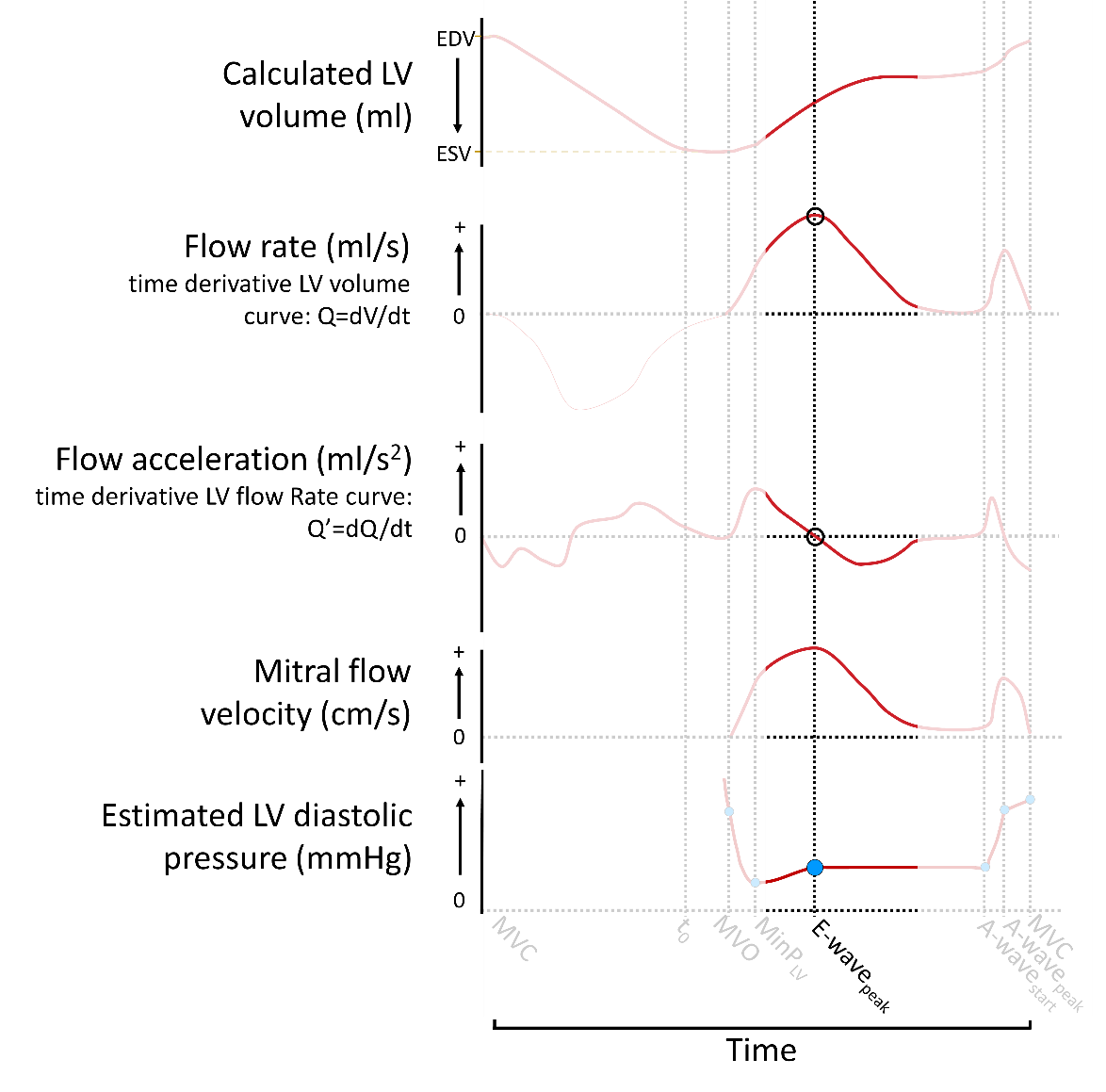


**Supplemental Figure S7:** Schematic of the estimation of pressure-time point of peak E-wave, with timing based on peak E flow rate (open circle).

1. **Start of A wave (P_A-start_, t_A-start_)**, see Figure S8: The estimates are $P_{\text{A-start}} (pre-A P\mathrm{LV})$, and t_A-start_ is the first time point after diastasis when d*V*/d*t* and d*Q*/d*t* turn positive ^(9)^.


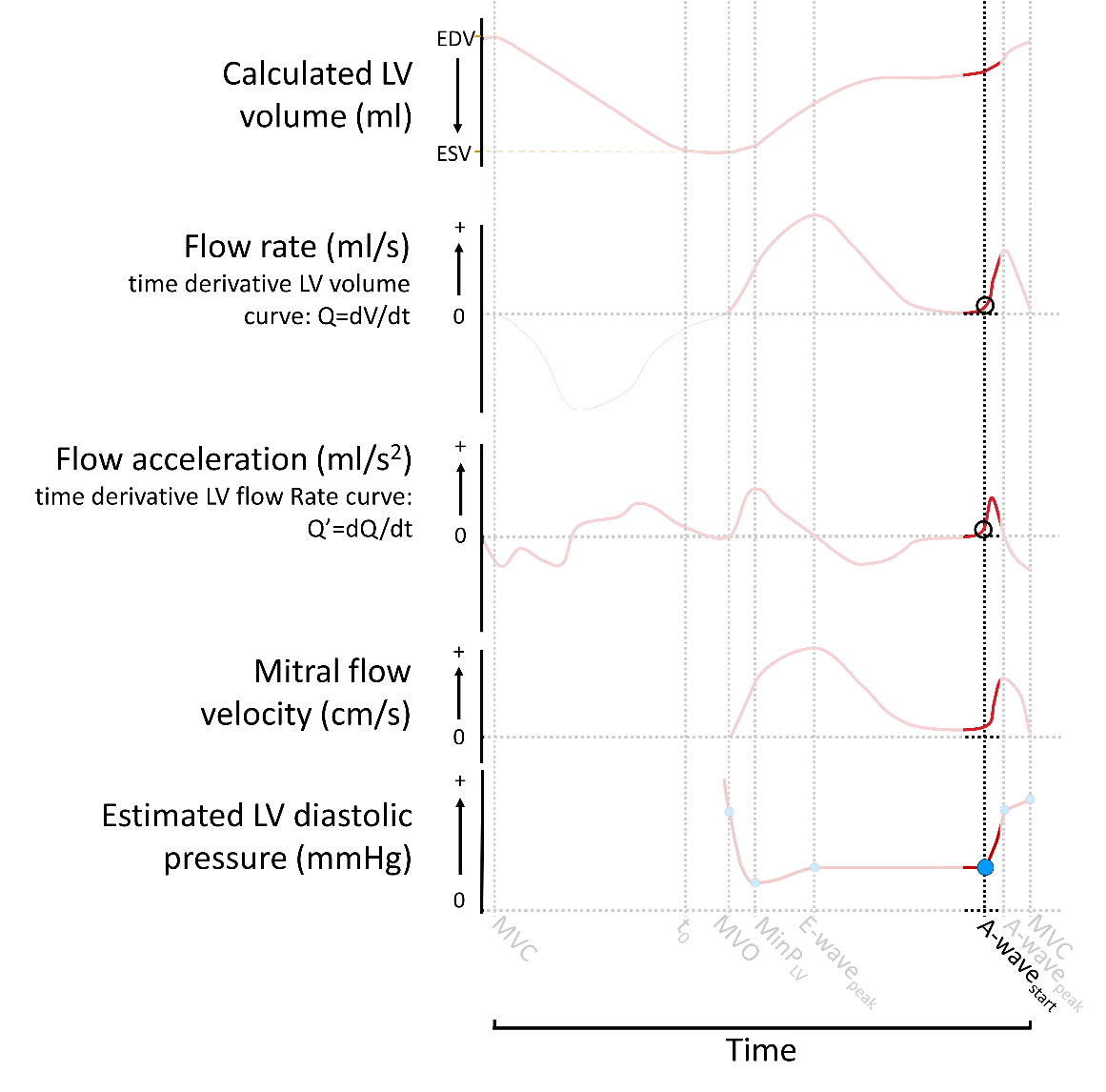


**Supplemental Figure S8:** Schematic of the estimation of pressure-time point of pre-A-wave (A-wave_start_), with timing based on onset of flow rate and flow acceleration (open circles)

1. **Peak of A-wave (P_A-peak_, t_A-peak_)**, see Figure S9: The pressure at this instant is estimated as the increment of pressure from the start of A-wave with two summands: the increase of passive filling (same linear pressure-volume model as used in peak E-wave) and the peak temporal acceleration of momentum over A-wave: $P_{\text{A-peak}}= P_{\text{A-start}}+{\Delta P}_{EDPVR during A acceleration}+max{\Delta P}_{\mathrm{MV}}$, where maximum ∆P_MV_ is given by equation S5 (the estimation of peak pressure drop follows the logic described in Supplementary Data D, and thus the instants of peak velocity and peak pressure drop might not be the same). And t_A-peak_ is the instant of peak velocity of the A-wave.


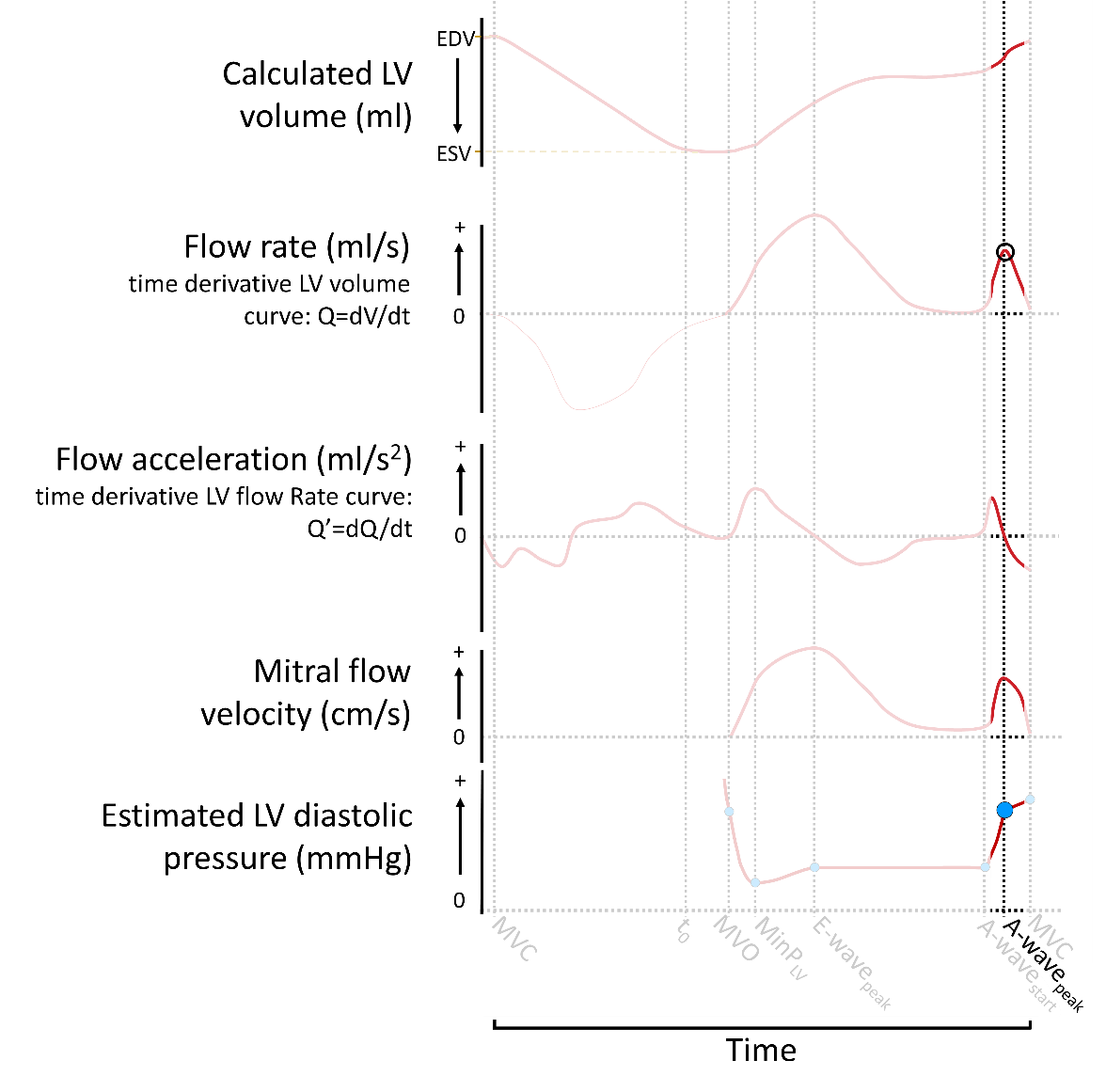


**Supplemental Figure S9**: Schematic of the estimation of pressure-time point of peak A-wave, with timing based on peak flow rate (open circle)

1. **Mitral valve closure (P_MVC_, t_MVC_),** see Figure S10: Similarly, the pressure at end-diastole/mitral valve closure (P_MVC_) is estimated based on the pressure due to the passive increase in pressure difference following the EDPVR (same as linear pressure-volume model as used in peak E-wave). The t_MVC_ is the instant of mitral valve closure (end-diastole).


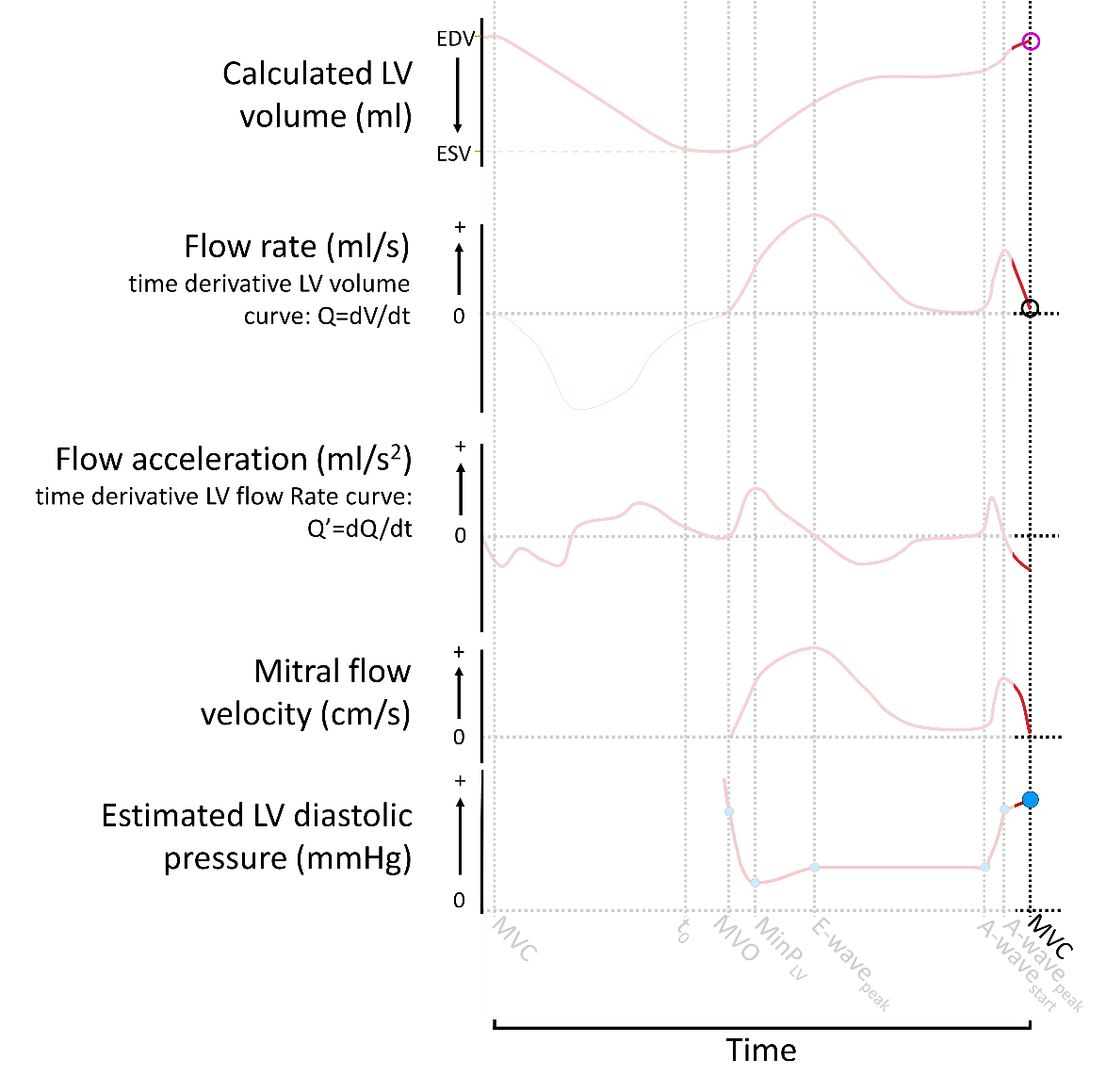


**Supplemental Figure S10**: Schematic of the estimation of pressure-time point of mitral valve closure (MVC), with timing based on end of the cycle accessed via LV volume trace or flow rate trace (open circles)

## Supplementary Data F: Sensitivity analysis of core input variables

### Introduction

A sensitivity analysis was performed to investigate the impact of errors in measurement of the core input variables on the major non-invasive estimates: minimum P_LV_, Pre-A P_LV_, end-diastolic P_LV_ and *τ*.

### Methods

The input variables considered were systolic pressure, isovolumetric relaxation time, peak A-wave peak velocity, E-wave peak velocity. An input error range of -20 % to +20 % is assumed over the baseline measurement of each patient. Five randomly selected patients are included in this study.

### Results

In general, all output variables were most sensitive to errors in IVRT, see Figure S11. Errors in A-wave measurements only produced minor changes in end-diastolic $P_{\mathrm{LV}}$ (respectively from -0.7 ± 0.2 mmHg to 0.9 ± 0.3 mmHg) and are thus not included in Figure S11.

### Conclusion

The reference key diastolic pressure points (minimum P_LV_, Pre-A P_LV_, end-diastolic P_LV_) and *τ* are reasonably robust to 20 % error in measurements in systolic pressure, IVRT and E-wave velocity.


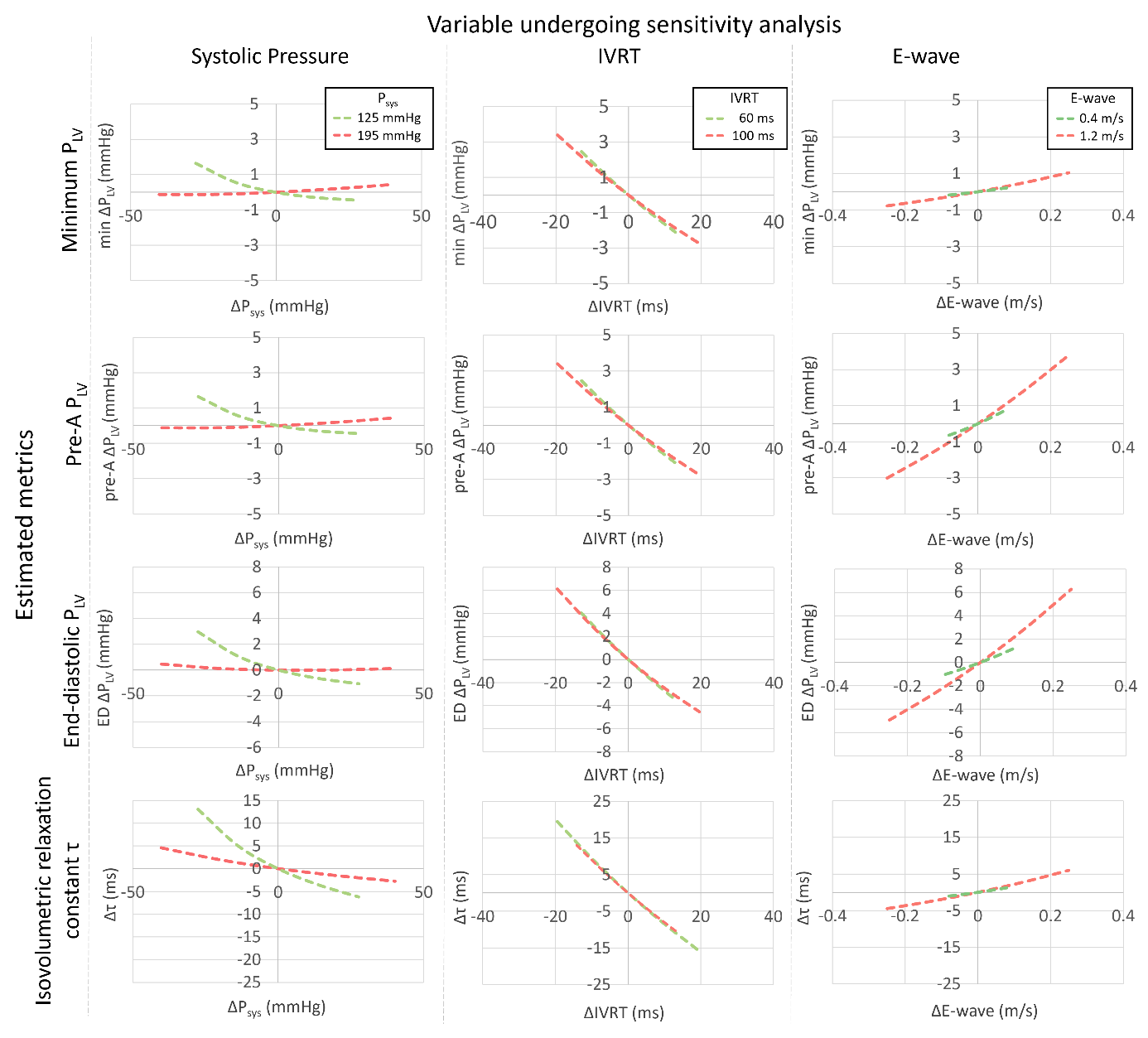


**Supplemental Figure S11: Sensitivity analysis of the main non-invasive diastolic pressure estimates.** In this analysis it is studied the impact of the core input variables variance on the key estimated metrics. The testing consisted in calculating the variability introduced in the estimates by a variation of each core input variable variation of ± 20 %. The variability range was selected in order to exacerbate the variability existing in standard clinical measurements. Each line represents one patient analysed, with a given measured input variable that represents an extreme value (lower value: green, higher value: red) for the respective variable. The non-invasive estimates tested were the diastolic left ventricular pressure (P_LV_) markers: Minimum P_LV_, pre-A P_LV_ and end-diastolic P_LV_ (ED P_LV_), and the isovolumetric relaxation constant τ. The input variables considered were the systolic pressure (P_sys_), the isovolumetric relaxation time (IVRT) and the transmitral E-wave velocity.

## References

1. Nagueh SF, Smiseth OA, Appleton CP, Byrd BF, Dokainish H, Edvardsen T, et al. Recommendations for the evaluation of left ventricular diastolic function by echocardiography: an update from the American Society of Echocardiography and the European Association of Cardiovascular Imaging. Eur J Echocardiogr 2016;17:1321-60.

2. Smiseth OA, Donal E, Boe E, Ha J-W, Fernandes JF, Lamata P. Phenotyping heart failure by echocardiography: imaging of ventricular function and haemodynamics at rest and exercise. European Heart Journal-Cardiovascular Imaging 2023;24:1329-42.

3. Smiseth OA, Morris DA, Cardim N, Cikes M, Delgado V, Donal E, et al. Multimodality imaging in patients with heart failure and preserved ejection fraction: an expert consensus document of the European Association of Cardiovascular Imaging. Eur Heart J Cardiovasc Imaging 2022;23:e34-e61.

4. Chubuchny V, Pugliese NR, Taddei C, Poggianti E, Spini V, Barison A, et al. A novel echocardiographic method for estimation of pulmonary artery wedge pressure and pulmonary vascular resistance. ESC Heart Failure. 2021;8(2):1216-29.

5. Russell K, Eriksen M, Aaberge L, Wilhelmsen N, Skulstad H, Remme EW, et al. A novel clinical method for quantification of regional left ventricular pressure-strain loop area: a non-invasive index of myocardial work. Eur Heart J 2012;33:724-33.

6. Yoshida T, Ohte N, Narita H, Sakata S, Wakami K, Asada K, et al. Lack of inertia force of late systolic aortic flow is a cause of left ventricular isolated diastolic dysfunction in patients with coronary artery disease. J Am Coll Cardiol 2006;48:983-91.

7. Smiseth OA, Steine K, Sandbaek G, Stugaard M, Gjolberg T. Mechanics of intraventricular filling: study of LV early diastolic pressure gradients and flow velocities. Am J Physiol 1998;275:H1062-9.

8. Weiss JL, Frederiksen JW, Weisfeldt ML. Hemodynamic determinants of the time-course of fall in canine left ventricular pressure. J Clin Invest 1976;58:751-60.

9. Rahimtoola SH, Loeb HS, Ehsani A, Sinno MZ, Chuquimia R, Lal R, et al. Relationship of pulmonary artery to left ventricular diastolic pressures in acute myocardial infarction. Circulation 1972;46:283-90.

10. Yamamoto K, Nishimura RA, Redfield MM. Assessment of mean left atrial pressure from the left ventricular pressure tracing in patients with cardiomyopathies. Am J Cardiol 1996;78:107-10.

11. Hasegawa H, Little WC, Ohno M, Brucks S, Morimoto A, Cheng HJ, et al. Diastolic mitral annular velocity during the development of heart failure. J Am Coll Cardiol 2003;41:1590-7.

12. Ishida Y, Meisner JS, Tsujioka K, Gallo JI, Yoran C, Frater RW, et al. Left ventricular filling dynamics: influence of left ventricular relaxation and left atrial pressure. Circulation 1986;74:187-96.

13. Yamamoto K, Masuyama T, Tanouchi J, Uematsu M, Doi Y, Naito J, et al. Importance of left ventricular minimal pressure as a determinant of transmitral flow velocity pattern in the presence of left ventricular systolic dysfunction. J Am Coll Cardiol 1993;21:662-72.

14. Thomas JD, Flachskampf FA, Chen C, Guererro JL, Picard MH, Levine RA, et al. Isovolumic relaxation time varies predictably with its time constant and aortic and left atrial pressures: implications for the noninvasive evaluation of ventricular relaxation. Am Heart J. 1992;124(5):1305-13.

15. Scalia GM, Greenberg NL, McCarthy PM, Thomas JD, Vandervoort PM. Noninvasive assessment of the ventricular relaxation time constant (τ) in humans by Doppler echocardiography. Circulation. 1997;95(1):151-5.

16. Cheng CP, Igarashi Y, Little WC. Mechanism of augmented rate of left ventricular filling during exercise. Circ Res 1992;70:9-19.

17. Flachskampf FA, Rodriguez L, Chen C, Guerrero JL, Weyman AE, Thomas JD. Analysis of mitral inertance: a factor critical for early transmitral filling. J Am Soc Echocardiogr. 1993;6(4):422-32.

18. Greenberg NL, Vandervoort PM, Thomas JD. Instantaneous diastolic transmitral pressure differences from color Doppler M mode echocardiography. Am J Physiol 1996;271:H1267-76.

19. Firstenberg MS, Vandervoort PM, Greenberg NL, Smedira NG, McCarthy PM, Garcia MJ, et al. Noninvasive estimation of transmitral pressure drop across the normal mitral valve in humans: importance of convective and inertial forces during left ventricular filling. J Am Coll Cardiol 2000;36:1942-9.

20. Holen J, Aaslid R, Landmark K, Simonsen S. Determination of pressure gradient in mitral stenosis with a non-invasive ultrasound Doppler technique. Acta Med Scand 1976;199:455-60.

21. Donati F, Myerson S, Bissell MM, Smith NP, Neubauer S, Monaghan MJ, et al. Beyond Bernoulli: Improving the Accuracy and Precision of Noninvasive Estimation of Peak Pressure Drops. Circ Cardiovasc Imaging 2017;10:e005207.

22. Akerman AP, Porumb M, Scott CG, Beqiri A, Chartsias A, Ryu AJ, et al. Automated echocardiographic detection of heart failure with preserved ejection fraction using artificial intelligence. JACC: Advances 2023;2:100452.

23. Lee E, Ito S, Miranda, Lopez-Jimenez F, Kane GC, Asirvatham SJ, WR et al. Artificial intelligence-enabled ECG for left ventricular diastolic function and filling pressure. NPJ Digit Med 2024;7:4.

24. Corral-Acero J, Margara F, Marciniak M, Rodero C, Loncaric F, Feng Y, et al. The 'Digital Twin' to enable the vision of precision cardiology. Eur Heart J 2020;41:4556-64.

25. Courtois MA, Sandor J, Kovacs J, Ludbrook PA. Physiological early diastolic intraventricular gradient is lost during acute myocardial ischemia. *Circulation.* 1990;*81*:1688–1696.

26. Steine K, Stugaard M, Smiseth OA. Mechanisms of retarded apical filling in acute ischemic left ventricular failure. Circulation. 1999 Apr 20;99(15):2048-54.

27. Ribic D, Remme EW, Smiseth OA, Massey RJ, Eek CH, Kvitting J-PE, et al. Non-invasive myocardial work in aortic stenosis–validation and improvement of left ventricular pressure estimation. European Heart Journal-Cardiovascular Imaging 2023:jead227.

28. Udelson JE, Bacharach SL, Cannon RO 3rd, Bonow RO. Minimum left ventricular pressure during beta-adrenergic stimulation in human subjects. Evidence for elastic recoil and diastolic "suction" in the normal heart. Circulation. 1990 Oct;82(4):1174-82.

29. Opdahl A, Remme EW, Helle-Valle T, Lyseggen E, Vartdal T, Pettersen E, et al. Determinants of left ventricular early-diastolic lengthening velocity: independent contributions from left ventricular relaxation, restoring forces, and lengthening load. Circulation. 2009;119(19):2578-86.

30. Courtois M, Fattal PG, Kovacs SJ, Jr., Tiefenbrunn AJ, Ludbrook PA. Anatomically and physiologically based reference level for measurement of intracardiac pressures. Circulation. 1995;92(7):1994-2000.

31. Grossmann W. “Pressure Measurement”. In Grossmann W and Baim DS (eds.), Cardiac Catheterization, Angiography and Intervention (4^th^ edition). Lea & Febiger, Philadelphia 1991. Pages 123-142.

32. Smiseth OA, Thompson CR, Lohavanichbutr K, Ling H, Abel JG, Miyagishima RT, et al. The pulmonary venous systolic flow pulse--its origin and relationship to left atrial pressure. J Am Coll Cardiol 1999;34:802-9.

33. Paulus WJ, Tschöpe C, Sanderson JE, Rusconi C, Flachskampf FA, Rademakers FE, et al. How to diagnose diastolic heart failure: a consensus statement on the diagnosis of heart failure with normal left ventricular ejection fraction by the Heart Failure and Echocardiography Associations of the European Society of Cardiology. Eur Heart J 2007;28:2539-50.

34. Myreng Y, Smiseth OA. Assessment of left ventricular relaxation by Doppler echocardiography. Comparison of isovolumic relaxation time and transmitral flow velocities with time constant of isovolumic relaxation. Circulation 1990;81:260-6.

35. Inoue K, Khan FH, Remme EW, Ohte N, García-Izquierdo E, Chetrit M, et al. Determinants of left atrial reservoir and pump strain and use of atrial strain for evaluation of left ventricular filling pressure. European Heart Journal-Cardiovascular Imaging 2022;23:61-70.

36. Schultz MG, Picone DS, Armstrong MK, Black JA, Dwyer N, Roberts-Thomson P, et al. Validation Study to Determine the Accuracy of Central Blood Pressure Measurement Using the Sphygmocor Xcel Cuff Device. Hypertension 2020;76:244-50.

37. Plankey MW, Stevens J, Flegal KM, Rust PF. Prediction equations do not eliminate systematic error in self-reported body mass index. Obes Res 1997;5:308-14.

38. Lamata P, Pitcher A, Krittian S, Nordsletten D, Bissell MM, Cassar T, et al. Aortic relative pressure components derived from four-dimensional flow cardiovascular magnetic resonance. Magn Reson Med 2014;72:1162-9.

39. Donati F, Figueroa CA, Smith NP, Lamata P, Nordsletten DA. Non-invasive pressure difference estimation from PC-MRI using the work-energy equation. Med Image Anal 2015;26:159-72.

40. Remme EW, Lyseggen E, Helle-Valle T, Opdahl A, Pettersen E, Vartdal T, et al. Mechanisms of preejection and postejection velocity spikes in left ventricular myocardium: interaction between wall deformation and valve events. Circulation 2008;118:373-80.

41. Ohno M, Cheng CP, Little WC. Mechanism of altered patterns of left ventricular filling during the development of congestive heart failure. Circulation 1994;89:2241-50.

42. Little WC, Ohno M, Kitzman DW, Thomas JD, Cheng CP. Determination of left ventricular chamber stiffness from the time for deceleration of early left ventricular filling. Circulation 1995;92:1933-9.

43. Zile MR, Baicu CF, Gaasch WH. Diastolic heart failure--abnormalities in active relaxation and passive stiffness of the left ventricle. N Engl J Med 2004;350:1953-9.25.
